# FAK inhibition alone or in combination with adjuvant therapies reduces cancer stem cell activity

**DOI:** 10.1038/s41523-021-00263-3

**Published:** 2021-05-28

**Authors:** Simon Timbrell, Hosam Aglan, Angela Cramer, Phil Foden, David Weaver, Jonathan Pachter, Aoife Kilgallon, Robert B. Clarke, Gillian Farnie, Nigel J. Bundred

**Affiliations:** 1 Breast Biology Group, Manchester Breast Centre, Oglesby Cancer Research Building, University of Manchester, Manchester, UK; 2 Cancer Stem Cell Research, Oglesby Cancer Research Building, University of Manchester, Manchester, UK; 3 Department of Oncology Cytogenetics, The Christie Foundation Trust, Manchester, UK; 4 Academic Surgery, University of Manchester, Manchester University NHS Foundation Trust, Manchester, UK; 5 Verastem Oncology, Needham, MA USA; 6 Present Address: Farnie Lab, Nuffield Department of Orthopaedics, Rheumatology and Musculoskeletal sciences, Botnar Research Centre, NDORMS, University of Oxford, Oxford, UK

**Keywords:** Breast cancer, Target identification, Cancer stem cells

## Abstract

Cancer stem-like cells (CSC) contribute to therapy resistance and recurrence. Focal adhesion kinase (FAK) has a role in CSC regulation. We determined the effect of FAK inhibition on breast CSC activity alone and in combination with adjuvant therapies. FAK inhibition reduced CSC activity and self-renewal across all molecular subtypes in primary human breast cancer samples. Combined FAK and paclitaxel reduced self-renewal in triple negative cell lines. An invasive breast cancer cohort confirmed high FAK expression correlated with increased risk of recurrence and reduced survival. Co-expression of FAK and CSC markers was associated with the poorest prognosis, identifying a high-risk patient population. Combined FAK and paclitaxel treatment reduced tumour size, Ki67, ex-vivo mammospheres and ALDH+ expression in two triple negative patient derived Xenograft (PDX) models. Combined treatment reduced tumour initiation in a limiting dilution re-implantation PDX model. Combined FAK inhibition with adjuvant therapy has the potential to improve breast cancer survival.

## Introduction

Breast cancer affects 1 in 8 women and there remains a need to improve survival, particularly within triple negative invasive breast cancer. Evidence indicates cancer stem-like cells (CSC), defined by their ability to self-renew and form a heterogeneous tumour are responsible for tumour initiation, maintenance, and metastasis^1^. In breast cancer CSC have been shown to be more resistant to radiotherapy^2^, endocrine^3^, and chemotherapy^4^. The ability of breast CSC to preferentially survive these treatments alongside their ability to self-renew and initiate recurrence has led to the search for anti-cancer stem cell agents^5^.

One potential target is focal adhesion kinase (FAK). FAK is classically known for its role in integrin related signalling at focal adhesions and its association with cell adhesion, survival, and metastasis^6^. High FAK expression was first associated with an invasive phenotype in breast cancer over 20 years ago^7^, and a meta-analysis across all solid cancers demonstrated that high FAK expression was associated with reduced survival^8^. Studies have also demonstrated that high FAK expression is associated with a triple negative phenotype and metastasis, with some showing a correlation with reduced breast cancer survival (Supplementary Table 1)^9–11^.

FAK consists of a central catalytic kinase domain, an N-terminal 4.1-erzin-radixin-moesin domain, a carboxy-terminal and a focal adhesion targeting domain. Each of these structural domains plays a different role although their full functions are yet to be elucidated^6^. The best characterised mechanism of FAK activation involves integrin receptor clustering upon binding of cells to the extracellular matrix leading to autophosphorylation at pTyr397. However, there are other phosphorylation sites outside of this activation loop which are involved with different aspects of FAKs role in tumour growth and metastasis^6^. FAK expression and active pTyr397 FAK has been shown to regulate CSC activity within breast cancer^12^. FAK ablation decreased the number of CSC identified using cell surface markers CD24+/CD29+/CD61+ or ALDH+ expression in a MMTV-PyMT mouse model^13^. Studies inhibiting FAK, reducing pTyr397, within invasive breast cancer and Ductal Carcinoma in Situ (DCIS) demonstrate decreased CSC activity and therapy resistance, which was associated with a reduction Wnt signalling^12,14^.

Breast cancer is currently managed by surgery, followed by multiple adjuvant therapies including radiotherapy, endocrine therapy, anti-Her2 agents, and chemotherapy. The addition of FAK inhibition is reported to overcome resistance to endocrine^15^ and chemotherapy^16^, as measured by tumour growth. This study investigated whether FAK inhibition in combination with appropriate standard adjuvant therapy can reduce tumour growth but more importantly CSC activity/tumour initiation. In addition, we used an invasive breast cancer cohort to investigate if high expression of FAK correlated with known CSC markers, ITGα6 and ALDH1.

## Results

### pTyr397FAK expression is higher in triple negative breast cancer cell lines and ALDH^+^ CSC population

**Fig. 1:**
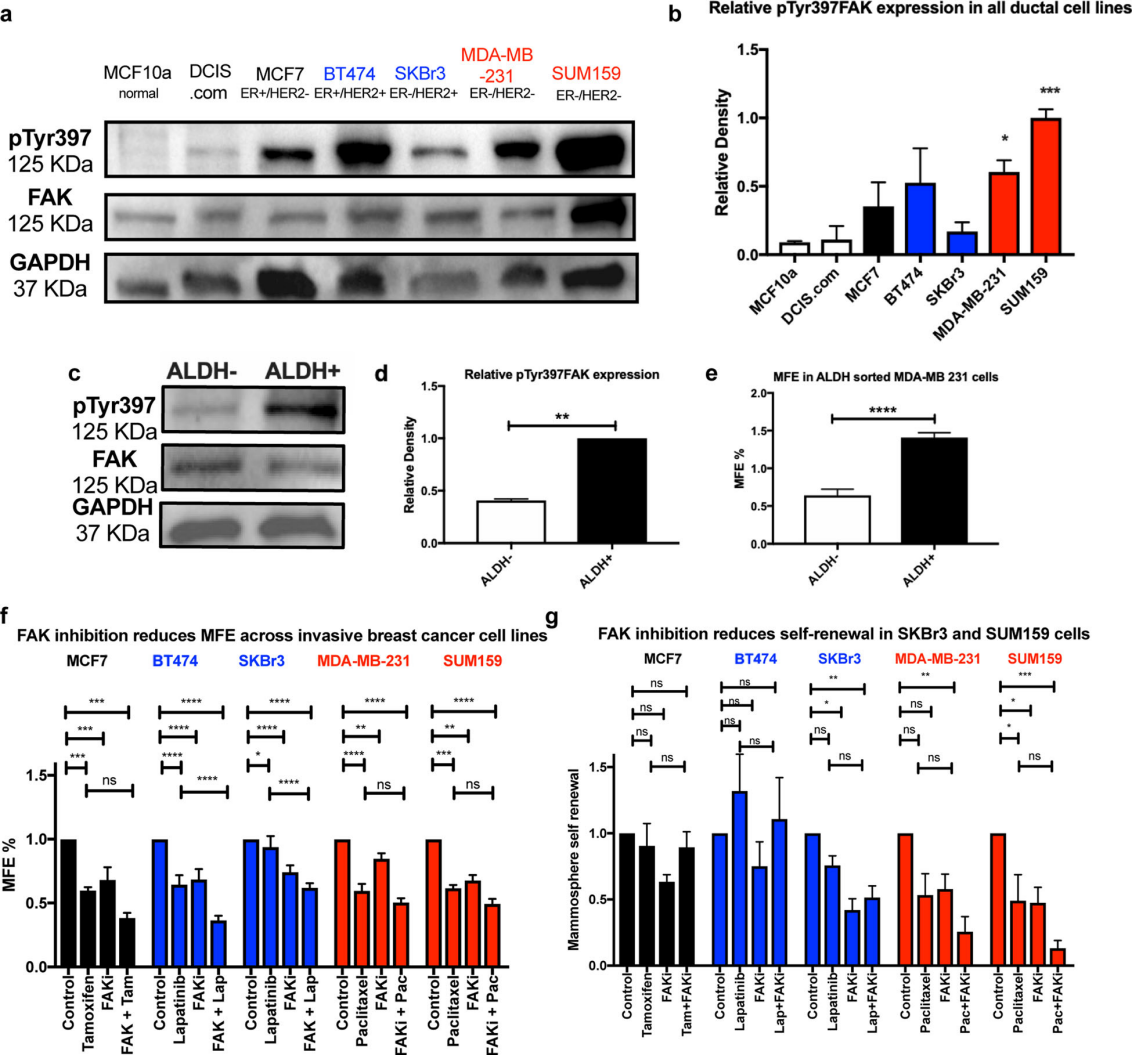
pTyr397FAK expression is higher in triple negative cell lines and ALDH^+^ cells. FAK inhibition in combination with adjuvant therapies reduces CSC activity. **a** Representative Western blot demonstrating baseline expression of pTyr397FAK, FAK, and GAPDH in breast cell lines including invasive carcinoma cell lines reflecting all molecular phenotypes. **b** Illustrative plot of relative density of pTyr397FAK to FAK, with relative density of pTyr397FAK to FAK measured and corrected for GAPDH. pTyr397FAK expression in normal ductal cell line MCF10a used as comparison. One-way ANOVA with post hoc Dunnett’s test (*n* = 2 for MCF10a and DCIS.com and *n* = 3 for IDC cell lines). **c** Representative Western blot demonstrating pTyr397FAK expression in ALDH^+^ and ALDH^−^ MDA-MB-231 cells with illustrative plot of relative densities shown in (**d**) (*n* = 3). **e** ALDH^+^ cells have increased primary MFE compared to ALDH^−^ expressing cells. Both D&E analysed using unpaired two tailed *t*-test. **f** FAK inhibition with VS4718 0.5 μM reduced primary MFE as a single agent therapy across all cell lines. This was evaluated alongside 1 μM of Tamoxifen in MCF7, 0.1 μM of Lapatinib in BT474 and SKBr3 cells and 0.1 μM Paclitaxel in MDA-MB-231 and SUM159 cells. Each experiment had a minimum of five biological repeats and six technical replicates. **g** FAK inhibition reduced mammosphere self-renewal in SKBr3 and SUM159 cells when used as monotherapy and in MDA-MB-231 cells when combined with Paclitaxel. All error bars are mean + SEM. Two-way ANOVA with post hoc Tukey’s test (ns not significant, *p < 0.05, **p < 0.01, ***p < 0.001, ***p < 0.0001).

We evaluated FAK expression across a range of invasive ductal cell lines to determine if high FAK or pTyr397FAK expression was associated with a particular molecular phenotype. pTyr397FAK expression was highest in the triple negative cell lines; MDA-MB-231 and SUM159 (Fig. 1a, b). All invasive ductal carcinoma cell lines expressed higher levels of FAK and pTyr397FAK when compared to the normal mammary (MCF10a) and pre-invasive (DCIS.com) cell lines (Fig. 1a, b). To determine if FAK expression was higher in CSC population as opposed to bulk tumour cells, we utilised flow cytometry to isolate ALDH^+^ expressing cells in MDA-MB-231s (see an example of ALDH gating in supplementary figure 1g). Western blot analysis demonstrated that ALDH^+^ cells express a 2.5-fold increase in pTyr397FAK compared to ALDH^−^ cells (Fig. 1c, d). We confirmed that ALDH^+^ population were enriched for CSC activity showing increased mammosphere forming efficiency of 1.41 ± 0.06% compared to 0.64 ± 0.08%, *p* < 0.0001 in ALDH^−^ cells (Fig. 1e). Although pTyr397FAK was elevated in all invasive breast cancer cell lines, evidence of increased pTyr397FAK within ALDH^+^ cells suggest FAK may be a promising target in triple negative breast cancers, where there are no current targeted therapies.

### FAK inhibition reduces mammosphere formation across all molecular phenotypes and improves mammosphere reduction in combination with Lapatinib in HER2+ cell lines

We investigated whether therapy resistant CSC populations could be reduced with FAK inhibitor, VS4718, and if combination treatment with adjuvant therapies would be more effective. Using the mammosphere assay, which measures changes in CSC activity in vitro, we investigated the effect of FAK inhibition (VS4718) alone and in combination with standard adjuvant therapies across a panel of breast cancer cell lines; MCF7 (ER+/PR+/HER2−) VS4718 0.5 μM ± Tamoxifen 1 µm; BT474 (ER+/PR+/HER2+) and SKBr3 (ER−/PR−/HER2+) VS4718 0.5 μM ± 0.1 µM Lapatinib; MDA-MB-231 and SUM159 (ER−/PR−/HER2−) VS4718 0.5 μM ± 0.1 µM Paclitaxel. FAK inhibition significantly reduced primary mammosphere formation efficiency across all molecular phenotypes ranging from 16% in MDA-MB-231 cells to 39% in SUM159 cells (Fig. 1f). When comparing combination treatment to adjuvant therapy alone (Tamoxifen, Lapatinib, or Paclitaxel) additional reductions in mammosphere formation were only significant in HER2+ve cell lines (BT474 and SKBR3).

### FAK inhibition reduces self-renewal in SKBr3 (HER2+) and SUM159 (triple negative) cell lines and in combination with Paclitaxel in MDA-MB-231 (triple negative) cells

To investigate the effects of treatments on a key CSC characteristic, self-renewal, primary mammospheres generated during the treatments described above were disaggregated to single cell suspensions and re-seeded for secondary mammosphere formation without further treatment. These experiments are analogous to in vivo limiting dilution assays to determine if treatment of the primary tumour has long lived effects to reduce tumour initiation. FAK inhibition alone (0.5 µM VS4718) significantly reduced secondary mammosphere formation/self-renewal by 57.94 ± 10.93% and 52.48 ± 15.20% in SKBr3 and SUM159 cell lines, respectively (Fig. 1g). In the triple negative MDA-MB-231 and SUM159 cell lines combined treatment of Paclitaxel (0.1 µM) and VS4718 (0.5 µM) was sufficient to significantly reduce self-renewal compared to control by 74.37 ± 19.92% and 86.82 ± 15.52%, respectively (Fig. 1g). Our data demonstrates FAK inhibition alone reduces CSC activity (primary mammospheres) in all molecular subtypes, however combination studies specifically show reductions in self-renewal after Lapatinib and Paclitaxel in Her2+ and triple negative cells.

### FAK inhibitor VS4718 reduces pTyr397FAK and mammosphere forming efficiency in a dose dependent manner in triple negative cell lines

**Fig. 2:**
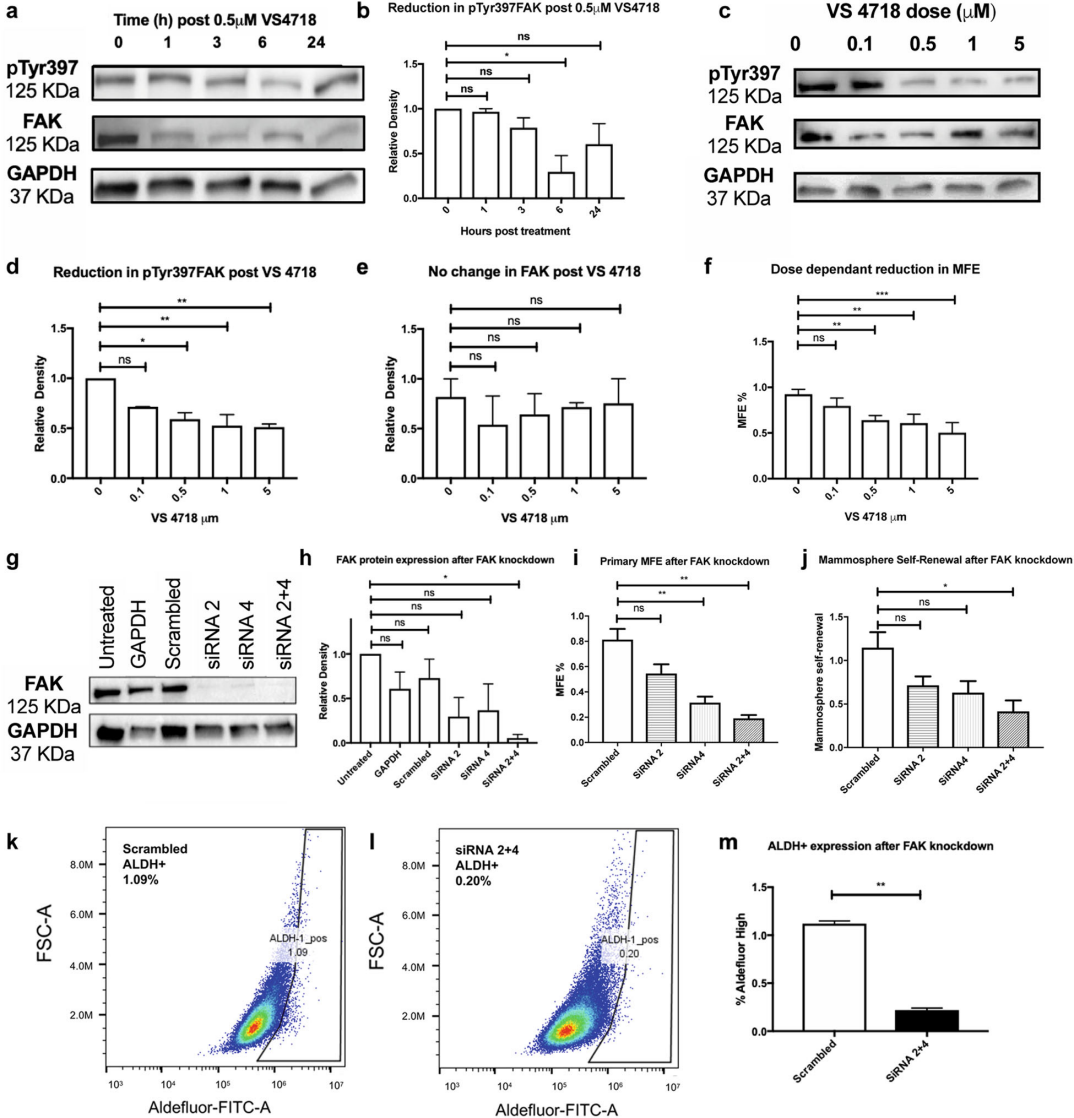
FAK inhibition and SiRNA knockdown reduces CSC activity in MDA-MB-231 cells. **a** Representative Western blot demonstrating that FAK inhibition with VS4718 resulted in a time dependent reduction in pTyr397FAK with relative protein expression shown in (**b**) but not FAK (*n* = 2). **c** Representative Western blot demonstrating pharmacological FAK inhibition with VS4718 resulted in a dose dependent reduction in pTyr397FAK with relative protein expression shown in (**d**) but not (**e**) FAK (*n* = 2). **f** The dose dependent reduction in pTyr397FAK corresponded with a reduction in primary MFE. **g** FAK knockdown using SiRNA resulted in a decrease in FAK expression with relative FAK: GAPDH after SiRNA knockdown shown in (**h**) relative density plot which correlated with a reduction in (**i**) primary MFE and (**j**) mammosphere self-renewal. One-way ANOVA with post hoc Dunnett’s test. **k** Representative FACS plots in scrambled controls and (**l**) SiRNA knockdown. **m** Graph demonstrating that FAK knockdown using SiRNA constructs 2 and 4 reduced ALDH^+^ expression (*n* = 2). Unpaired two-tailed *t*-test. (ns not significant, *p < 0.05, **p < 0.01, ***p < 0.001, ***p < 0.0001). All experiments were *n* = 3 unless otherwise stated and error bars represent mean + SEM.

We further explored the effects VS4718 in MDA-MB-231 and SUM159, as there is need for targeted therapy for this aggressive breast cancer. VS4718 (0.5 µM) led to a reduction in pTyr397FAK expression in a time (Fig. 2a, b) and dose dependent manner (Fig. 2c, d). A significant reduction in pTyr397FAK was seen in both MDA-MB-231 and SUM159s (Fig. 2a, e and Supplementary Fig. 2a–c) at a dose of 0.5 µM and 6 h post-administration. FAK inhibition resulted in a dose dependent reduction in primary mammosphere forming efficiency (Fig. 2f and supplementary 2h), which correlated reduced pTyr397FAK. Levels of pTyr397 kinase activity correlated directly with CSC activity indicating this was a good therapeutic target.

### FAK SiRNA knockdown reduces total FAK expression, mammosphere formation, self-renewal, and ALDH^+^ population in triple negative cell lines

In order to confirm the reduction in CSC activity was via FAK and not off target we investigated changes in mammospheres and self-renewal after SiRNA knockdown of FAK (PTK2). FAK knockdown with two independent SiRNA (#2, #4) was successful and a combination of both SiRNA led to a significant reduction 94.58 ± 26.89% in FAK protein expression in MDA-MB-231s (Fig. 2g, h). Double SiRNA knockdown corresponded with a 76.50 ± 10.24%, *p* < 0.01 reduction in primary mammosphere formation (Fig. 2i) and a 63.66 ± 16.27%, *p* < 0.05 reduction in self-renewal (Fig. 2j). Similar results were seen in SUM159s (Supplementary Fig. 2d–h). FAK knockdown reduced ALDH^+^ expression in MDA-MB-231s from 1.12 ± 0.03% in scrambled SiRNA controls to 0.20 ± 0.02%, *p* < 0.01 (Fig. 2k–m). These data generated by specific FAK siRNA knockdown confirm the reduction in CSC activity after VS4718 treatment was principally caused by decreased FAK activity (pTyr397FAK) and not off target effects.

### FAK inhibition reduces primary and secondary mammosphere formation in patient samples across all molecular phenotypes

**Fig. 3:**
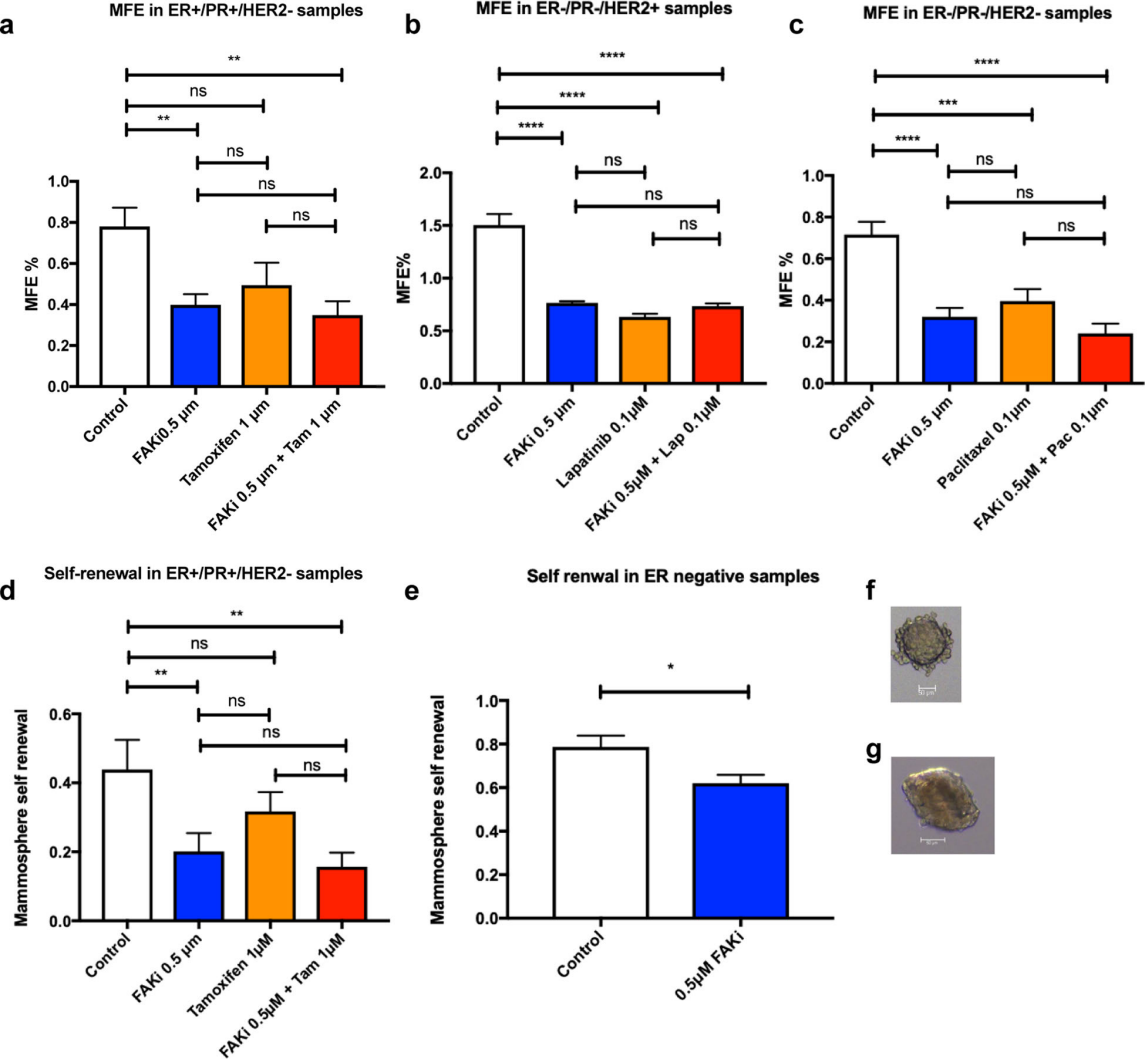
FAK inhibition reduces primary and secondary sphere formation across all molecular phenotypes in patient samples. **a** FAK inhibition with 0.5 μM VS4718 reduces primary mammosphere formation in 16 ER+/PR+/HER2− patient samples. **b** 0.5 μM VS4718 and 0.1 μM Lapatinib reduced primary MFE in four ER−/PR−/HER2+ patient samples. **c** 0.5 μM VS4718 and 0.1 μM Paclitaxel reduced primary MFE in six ER−/PR−/HER2− patient samples. Each patient had a minimum of six technical replicate values for MFE. **d** Graph demonstrating that FAK inhibition reduces mammosphere self-renewal in eight ER+/PR+/HER2− samples. Data in (**a**–**d**) analysed using Two-way ANOVA with post hoc Tukeys test. **e** Graph demonstrating that FAK inhibition in ER negative patient samples reduces mammosphere self-renewal. Unpaired two-tailed *t*-test. (ns not significant, *p < 0.05, **p < 0.01, ***p < 0.001, ***p < 0.0001). All error bars represent mean + SEM. Photomicrographs taken at ×40 magnification of (**f**) primary patient mammosphere after VS4718 treatment (**g**) secondary mammosphere in DMSO group with 50 µM scale bars.

The use of patient derived primary breast cancer samples allow our studies to be verified in early breast cancer cells and not cell lines derived from metastasis, in addition they are more representative of the response to treatment seen within a heterogenous breast cancer population. FAK inhibition reduced primary mammosphere formation in 16 ER+/PR+/HER2− samples from 0.78 ± 0.10% to 0.40 ± 0.10%, *p* < 0.01 (Fig. 3a), which did not improve in combination with Tamoxifen. In four ER−/PR−/HER2+ samples primary mammosphere formation was reduced from 1.51 ± 0.08% to 0.77 ± 0.08%, *p* < 0.001 after VS4718 inhibition, and to 0.63 ± 0.08%, *p* < 0.001 with Lapatinib alone. No additional combination effect was seen (Fig. 3b). In six ER−/PR−/HER2− patient samples, 0.5 µM VS4718 and Paclitaxel alone reduced primary mammosphere formation from 0.72 ± 0.07% to 0.32 ± 0.07%, *p* < 0.0001 and 0.40 ± 0.08%, *p* < 0.001, respectively with no combination effect (Fig. 3c).

FAK inhibition alone (0.5 µM) resulted in a 54.11 ± 14.40%, *p* < 0.01 reduction in self-renewal (secondary mammosphere formation) in eight ER+/PR+/HER2− patient samples. Tamoxifen treatment alone did not significantly reduce self-renewal or produce an additional combined effect (Fig. 3d). FAK inhibition alone reduced self-renewal by 22.52 ± 8.1%, *p* < 0.05 in 4 ER− patient samples (Fig. 3e). Images representative of primary and secondary patient derived mammospheres are shown in Fig. 3f, g, respectively. These data show FAK inhibition reduced self-renewal across all molecular phenotypes in early invasive breast cancer primary patient tissue compared to cell line data, where only Her2+ and triple negative were affected.

### High FAK is associated with poor prognosis

**Fig. 4:**
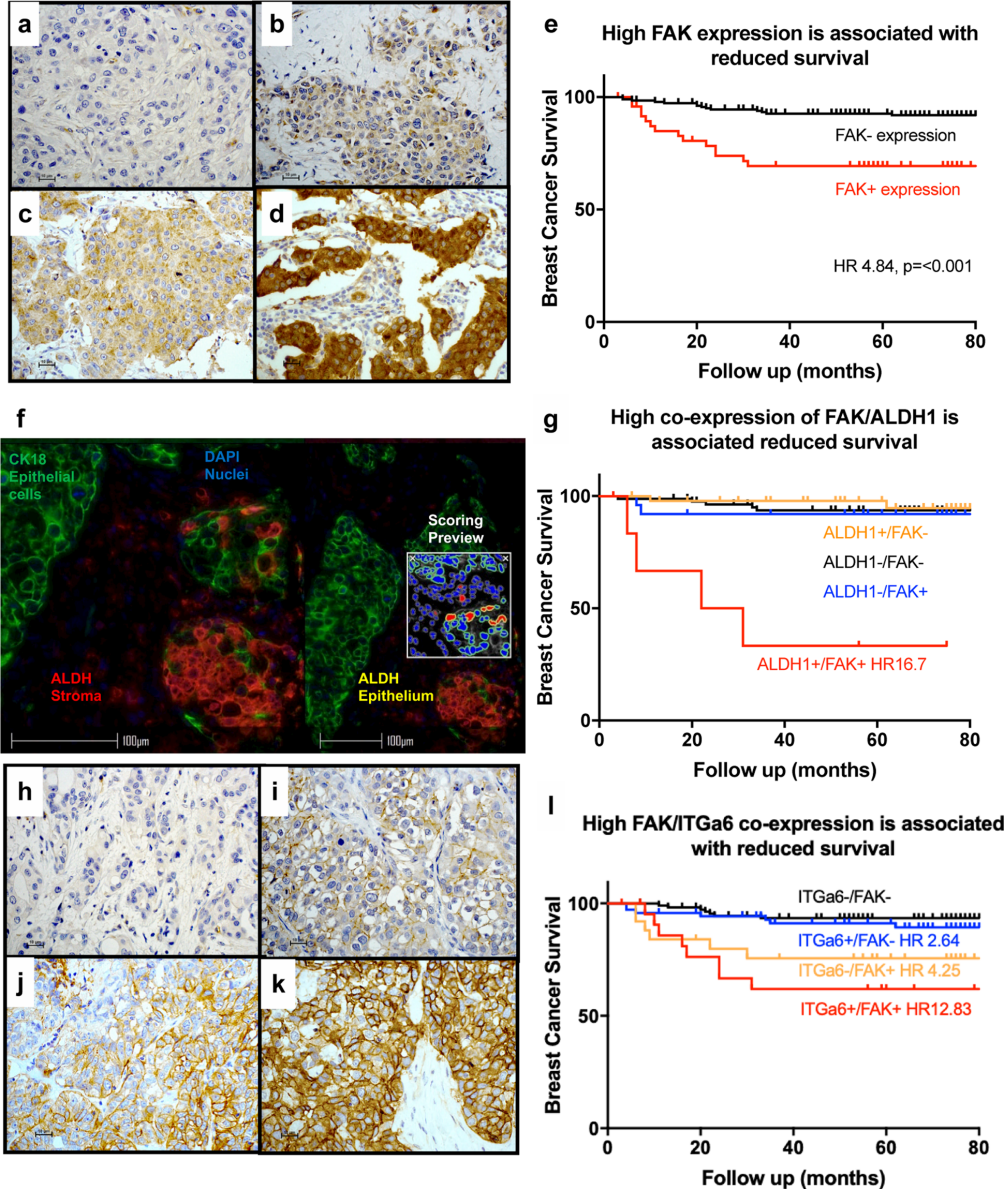
High FAK and high FAK/ALDH1 or FAK/ITGα6 expression is associated with reduced breast cancer survival. Representative photomicrographs of FAK staining taken at ×40 magnification with 10 µM scale bars demonstrating; **a** no staining, **b** weak staining, **c** moderate staining, and (**d**) high staining. High FAK expression was defined as high intensity staining in greater than 20% of epithelial cells as per Yom et al. criteria. **e** Kaplan–Meier demonstrating high FAK was associated with reduced survival, HR 4.84 (2.33–10.04, Cox-proportional regression *p* < 0.001). **f** Representative photomicrograph, with 100 µM scale bar of immunofluorescence staining with epithelial cells isolated using a CK18 antibody conjugated with a green fluorophore and ALDH1 conjugated to a red fluorophore. Automated analysis of dual staining was then undertaken using the HALO imaging system with a preview window at ×12 magnification demonstrating how the co-detection system worked. **g** Kaplan–Meier demonstrating high co-expression of FAK and ALDH1 (defined as greater than 5% epithelial positivity) is associated with an increased risk of breast cancer death (*n* = 162) HR 16.7 (5.21–38.05, Cox-proportional hazard regression *p* < 0.001). Representative photomicrographs of ITGα6 staining taken at ×40 magnification with 10 µM scale bar demonstrating; **h** no staining, **i** weak staining, **j** moderate staining, and (**k**) high staining. High ITGα6 was defined using Friedrich et als criteria of moderate or high intensity in greater than 5% of epithelial cells. **l** Kaplan–Meier demonstrating the association of high FAK and high ITGα6 expression with reduced survival (*n* = 230). HR 12.83 (4.43–37.13, Cox-proportional hazard regression *p* < 0.001).

We have shown FAK is important in regulating CSCs therefore we used a retrospective cohort of patients with invasive ductal carcinoma to determine if FAK expression was associated with outcome. High expression was defined using Yom et als criteria^17^ whereby high intensity staining (Fig. 4d) was present in greater than 20% of epithelial cells. See Fig. 4a–d for the range of FAK expression, with smooth muscle actin as an internal control for moderate staining (Fig. 4c). Low FAK was defined as any other staining intensity excluding High FAK criteria. High FAK was associated with reduced breast cancer survival (cox-proportional hazard regression ratio) HR 4.84, *p* ≤ 0.001, Fig. 4e. High FAK was associated with increased metastasis HR 3.02, *p* ≤ 0.001 and increased breast cancer recurrence HR 2.05, *p* = 0.005 (Supplementary Fig. 3a, b).

Phosphorylated Tyr397FAK expression was not associated with outcome in our cohort (Supplementary Fig. 3c, d). We demonstrated, with prospective breast cancer tissue collection that pTyr397FAK degraded in a time dependant manner (pre-fixation), whereas total FAK expression did not degrade (Supplementary Fig. 3e). Our cohort was retrospectively collected from formalin fixed paraffin embedded pathology blocks, where the time between surgery and tissue fixation was not recorded and is likely to vary between samples. This may account for the lack of association of pTyr397FAK with outcome.

### High ALDH1 and ITGα6 expression are both associated with poor clinical outcome

Since FAK regulates CSC activity, we investigated the expression of two CSC biomarkers; ALDH1^17^ and ITGα6^18^ alone and co-expressed with high FAK expression. We evaluated ALDH1 expression using a dual immunofluorescent staining protocol (Fig. 4f) with epithelial cells identified using a CK18 antibody conjugated to a green fluorophore and ALDH1 antibody conjugated with a red fluorophore. High epithelial ALDH1 expression (>5%) was associated with increased risk of breast cancer death HR 6.58, *p* = 0.003 (Supplementary Fig. 4a) and recurrence HR 2.21, *p* = 0.011 (Supplementary Fig. 4b, c). High expression of ITGα6 was defined using Friedrich et al.’s criteria^18^ (Fig. 4h–k). High ITGα6 was associated with ER negative disease and triple negative phenotype (Supplementary Fig. 4d). High expression of ITGα6 expression was associated with increased breast cancer death HR 2.23, *p* = 0.03 (Supplementary Fig. 4e) and metastasis HR 2.17, *p* = 0.008 (Supplementary Fig. 4f).

### High co-expression of FAK and CSC markers ALDH1 and ITGα6 is associated with the poorest prognosis

Co-expression of high FAK and ALDH1 was associated with an increased risk of breast cancer death HR 16.70, *p* =< 0.001. These breast cancer deaths occurred within the first 40 months (Fig. 4g). High FAK and high ITGα6 was associated with an increased risk of breast cancer death HR 12.83, *p* =< 0.001, Fig. 4l. This data further suggests the link between FAK expression and CSC activity and also identifies a high-risk patient population.

### In vivo FAK inhibition reduces tumour growth and Ki67 expression in two triple negative PDX models

**Fig. 5:**
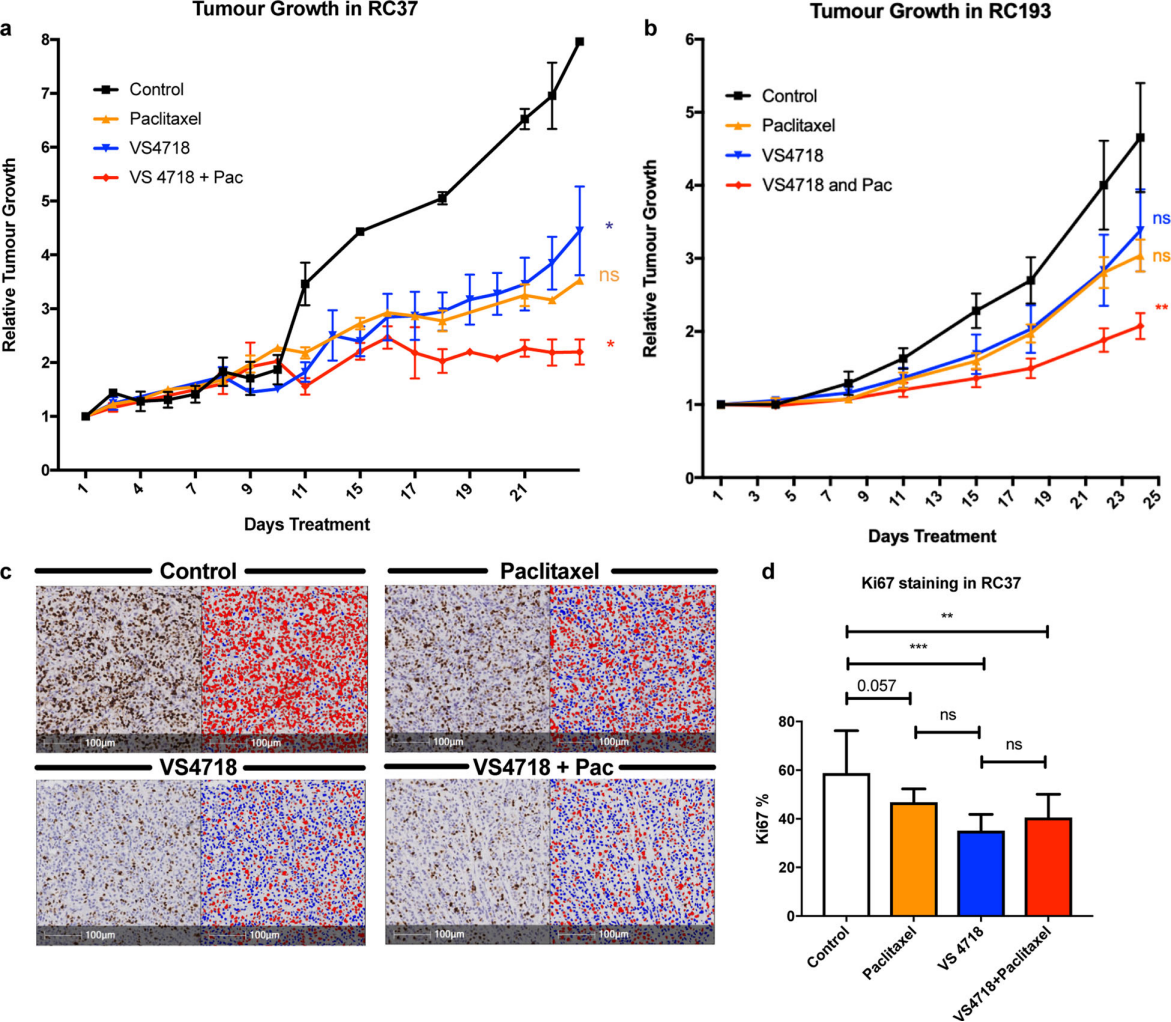
FAK inhibition reduces primary tumour growth and proliferation in triple negative PDX models. **a** Tumour growth curve demonstrating that 50 mg/kg per oral VS4718 on weekdays as monotherapy and combined weekly Paclitaxel 7.5 mg/kg via intraperitoneal injection reduces relative tumour growth compared to control mice in RC 37. **b** Tumour curve demonstrating that combined FAK and Paclitaxel reduced tumour growth relative to control in RC193. Two-way ANOVA with post hoc Dunnett’s test. **c**) Photomicrographs of Ki67 staining with associated 100 µm scale bars. Second column demonstrates the automated scoring system used by the HALO system to score the number of positive nuclei shown in red. **d** 0.5 μM VS4718 reduced Ki67 expression in RC37. Two-way ANOVA with post hoc Tukeys test (ns not significant, *p < 0.05, **p < 0.01, ***p < 0.001, ***p < 0.0001). All error bars represent mean + SEM.

To confirm our in vitro data we selected two triple negative patient derived xenograft (PDX) models RC37 and RC193 (Supplementary Fig. 5b–d). VS4718 50 mg/kg given twice daily via oral gavage led to a 60.24% ± 11.13, *p* = 0.010 and a 57.58% ± 14.22, *p* = 0.016 reduction tumour volume in RC37 and RC193 respectively (Fig. 5a, b). VS4718 treatment reduced proliferation marker Ki67 by 23.7% ± 12.1, *p* < 0.0001 compared to vehicle control (Fig. 5c, d). Combined VS4718 and Paclitaxel treatment did not reduce Ki67 expression further (mean reduction 18.3 ± 12.1%, *p* = 0.0022; Fig. 5d). There was a trend to suggest 7.5 mg/kg of Paclitaxel alone reduced Ki67 with a mean difference of 12.0% *p* = 0.057. This suggests VS4718 reduces the proliferation of bulk tumour cells as well as CSC activity.

### In vivo FAK inhibition in two triple negative PDX reduces ex vivo mammosphere formation, ALDH^+^ CSC population and tumour initiating capacity

**Fig. 6:**
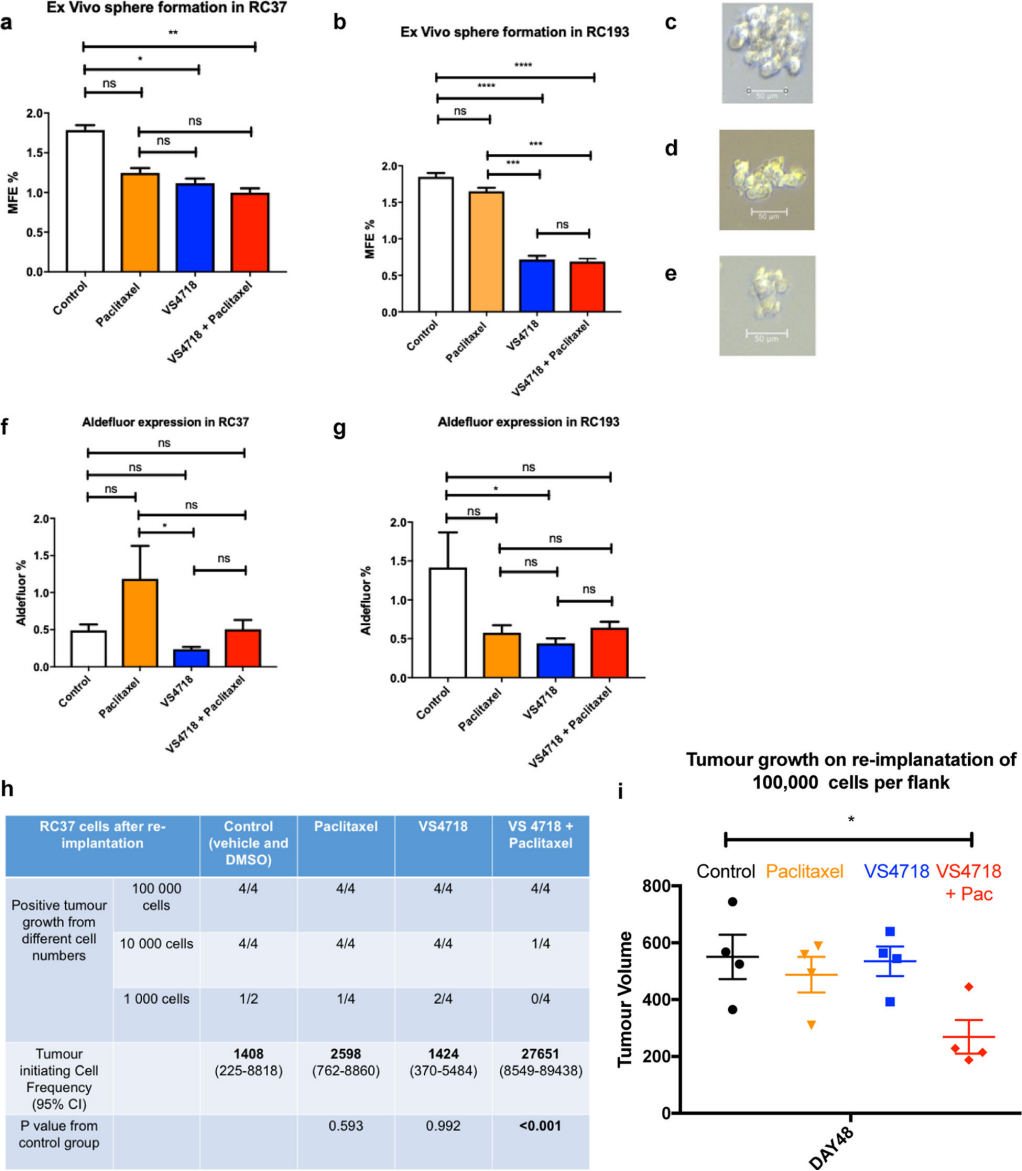
FAK inhibition reduces ex vivo mammosphere formation, ALDH^+^ expression and tumour initiating capacity. **a** Graph demonstrating that FAK inhibition reduced ex vivo MFE when used alone or with Paclitaxel in RC37. **b** Graph demonstrating that VS4718 reduced MFE relative to control and Paclitaxel treated mice in RC193. Photomicrograph taken at ×40 magnification and 50 µM scale bars of mammospheres isolated ex vivo from; **c** RC37 control group, **d** RC37 VS4718 treated group, and **e** RC193 control receiving group. **f** Graph demonstrating a reduction in ALDH^+^ expression in VS4718 treated mice as opposed to Paclitaxel treated mice in RC37. **g** Graph demonstrating that VS4718 reduced ALDH^+^ in RC193 relative to control mice. Data in (**a**, **b**, **f**, and **g**) analysed using Two-way ANOVA with post hoc Dunnett’s test. **h** FACS plots demonstrating the identification of epithelial staining of ALDH was identified using PDX model. General cells were selected, then live cells isolated using 7AAD. Mouse cells were then excluded using H2KD. For the Aldefluor assay the DEAB control was set at 0.1%. With ALDH^+^ expression in control mice and VS4718 treated mice in the RC193 model shown in the last two panels. **i** Limiting dilution table demonstrating that combined FAK inhibition and Paclitaxel treatment led to a reduction in tumour initiating capacity. Tumours were defined based on size greater than 100 mm^3^ at Day 60 and ELDA analysis performed using online tool at: http://bioinf.wehi.edu.au/software/elda. **j** Combined FAK inhibition and Paclitaxel treatment resulted in a reduction in tumour size in the 100,000 cells per flank transplantation on Day 48. Data are represented as mean + SEM. (One-way ANOVA analysis with post hoc Dunnett’s test; *p < 0.05).

To evaluate if the reduction in growth and proliferation during FAK inhibitor treatment corresponded with a decreased CSC activity, we isolated tumour cells from treated PDX tumours and measured ex vivo mammosphere forming ability (representative photomicrographs Fig. 6c–e), ALDH^+^ CSC population (Supplementary Fig. 1a–f) and tumour initiating capacity. In vivo administration of VS4718 decreased ex vivo mammosphere formation by 38% in RC37 from 1.79 to 1.12%, *p* = 0.005. Combined VS4718 and Paclitaxel resulted in a further reduction (45%) in ex vivo mammosphere formation to 0.99%, *p* < 0.001 (Fig. 6a). The second PDX model, RC193, showed similar results. VS4718 reduced ex vivo mammosphere formation by 61.1% from 1.85 to 0.71%, *p* < 0.001 and VS4718 alone resulted in a reduction in mammosphere formation relative to Paclitaxel from 1.65 to 0.72%, *p* < 0.001 (Fig. 6b). No change in ALDH^+^ CSC populations were seen in treated compared to control tumours in the RC37, but a reduction in ALDH^+^ CSC population was observed when comparing Paclitaxel (1.19%) to VS4718-treated PDX (0.24%, *p* = 0.033) (Fig. 6f). RC193 had a larger ALDH^+^ CSC population in control conditions (1.41%), here FAK inhibition resulted in a decrease in ALDH^+^ cells to 0.97%, *p* < 0.001 (Fig. 6g).

Cells from the treated tumours were also re-implanted into recipient mice at limiting dilution and tumour initiating capacity (TIC) calculated using the extreme limiting dilution assay (ELDA). PDX treated with a combination of VS4718 and Paclitaxel had a significant reduction in TIC with no tumours forming when 1000 cells were implanted into NSG mice (Fig. 6h). Even when 10,000 cells were implanted only one out of four tumours formed after 60 days compared to four out of four across the remaining groups (Fig. 6h). TIC frequency in control tumours was one in every 1408 cells, compared with one in every 27,651 within those tumours harvested from PDX receiving combined VS4718 and Paclitaxel treatment (*p* < 0.001, Fig. 6h). It is important to note that one mouse died in the control group (1000 cells) and if tumours were to have formed in this mouse, we anticipate that there may have been a significant reduction in TIC after VS4718 alone.

As well as reducing TIC on serial transplantation, combined VS4718 and Paclitaxel treatment significantly reduced tumour growth rate by 52.26% compared to control at day 48 when 100,000 cells were re-implanted, *p* < 0.05 (Fig. 6i). Thus, combined FAK inhibition and Paclitaxel treatment reduced TIC and the rate of subsequent re-growth which would improve long term survival.

## Discussion

FAK inhibition, via reduction in pTyr397FAK, reduced CSC activity and self-renewal across all molecular subtypes in primary human breast cancer samples. Our studies show that FAK inhibition in combination with adjuvant therapies, Lapatinib and Paclitaxel, improved reduction of CSC activity in Her2+ and triple negative breast cancer cell lines. Importantly, FAK and Paclitaxel combination studies demonstrate a reduction in the frequency of tumour initiating cells, alongside ex vivo mammosphere formation and ALDH^+^ expression, in a triple negative PDX.

In our retrospective case–control cohort, we demonstrate that high FAK expression, but not pTyr397FAK, was associated with reduced breast cancer survival and increased risk of recurrence, in keeping with a meta-analysis in solid cancers^8^. Unlike FAK expression, pTyr397FAK staining demonstrated degradation of pTyr397FAK, as seen with other phosphorylated proteins^19^ with increasing time to fixation. This suggests pTyr397FAK staining may not be reliable within retrospective cohorts and to validate pTyr397FAK expression in clinical specimens prospective studies should formalin fix tissue <1 h after excision^20^. High CSC marker expression alone (ALDH1 or ITGα6) was associated with a poor prognosis in our cohort as previously reported^18,21–23^. Co-expression of high FAK expression and high expression of either CSC marker (ALDH1 or ITGα6) is associated with a particularly poor prognosis identifying a high-risk patient population, which are likely to benefit from FAK inhibition.

High FAK expression has previously been shown in anoikis resistant CSC cell populations within DCIS models^14^ and FAK inhibition causes preferential cell death within ALDH^+^ MDA-MB-231 and SUM159 CSC populations^12^. Our study shows ALDH^+^ cells were enriched for mammosphere forming cells^21^ and expressed greater pTyr397FAK, suggesting FAK inhibition preferentially targets CSC-like cells. This is in agreement with our in vivo data showing FAK inhibition reduced CSC self-renewal, tumour initiation, and overall tumour proliferation. A limitation of our work is that we only evaluated the pTyr397 phosphorylation site, which has been studied extensively in previous work^6^ and its inhibition reduces CSC activity^12,14^. Although this phosphorylation site within the central kinase domain plays a key role in FAKs’ contribution to cell motility^6^, there are other phosphorylation sites and mechanisms of FAK activation. For example P53 is known to bind to the FERM domain of FAK, and nuclear FAK has been shown to regulate cell cycle progression via kinase independent mechanisms in a SCC model^6,24^.

Previous studies into the effects of FAK inhibition in primary patient tissue only include a small number of samples. Kolev et al evaluated three patient derived samples^12^, whereas this study uses 26 early invasive breast cancers, the largest evaluation of FAK inhibition in patient samples. FAK inhibition reduced in vitro CSC activity, measured by primary mammosphere formation, across all molecular phenotypes in both cell lines and patient samples. A reduction in primary mammosphere formation does not just measure a reduction in self-renewal, it is also affected by changes in apoptosis or proliferation^25^, leading to a reduced mammosphere size. Therefore, we specifically evaluated self-renewal by isolating single cells from primary mammospheres, treated with FAK inhibitor or control, and re-plated these cells for secondary mammospheres formation, with no further treatment. FAK inhibition reduced self-renewal in primary patient samples in all molecular subtypes, however two of the three ER− cell lines (SKBr3 and SUM159) had a reduction in self-renewal after FAK inhibition alone, which was not seen in either ER+ cell (MCF7 or BT474). The different self-renewal response within patient samples and cell lines may be attributed to their origin. Many of the cell lines utilised were cultured from metastatic tissue such as pleural aspirates and acquire genomic mutations as they are passaged, and may not reflect the primary breast tumour. This may explain the reduced sensitivity of cell lines to FAK inhibition compared to primary patient derived tissue which is more representative of early breast cancer. This was also demonstrated within our triple negative breast cancer cell lines, where FAK inhibition reduced self-renewal in the SUM159 cell line, which is established from a breast tumour primary but not in the MDA-MB-231 cell line which is established from a metastatic pleural aspirate. We propose that FAK inhibition may prove to be more beneficial in the early breast cancer setting than the metastatic setting where it is currently being investigated^26,27^.

Additional data generated through SiRNA knockdown of FAK in MDA-MB-231 cells also corroborate these suggestions, where a greater reduction primary mammosphere formation is observed and unlike with VS4718, self-renewal was also reduced. This is likely explained by the fact protein knockdown generally produces a bigger phenotypic effect and downregulation of associated signalling than drug inhibition of its active site leading to SiRNA but not VS4718 causing a reduction in the MDA-MB-231 cells.

Resistant breast tumour populations surviving in patients, as residual disease, after receiving conventional treatments, endocrine therapy (letrozole), or chemotherapy (docetaxel), are enriched for CSC tumour-initiating cell with mesenchymal features^28^. Elimination of this resistant population is key to improve long term survival in patients. In vitro investigations of FAK inhibition in combination with Tamoxifen, Lapatinib, and Paclitaxel, in ER+, Her2+, and triple negative breast cancers respectively, demonstrated additional reductions in CSC activity (primary mammosphere formation) with Lapatinib and Paclitaxel. In particular VS4718 and Paclitaxel also reduced self-renewal in triple negative primary breast cancer samples, showing the potential for decreasing the number of residual therapy resistant cells. Triple negative cell lines were shown to have elevated pTyr397FAK expression in agreement with clinically derived data indicating high FAK expression correlates with a triple negative phenotype, p53 loss, lymphovascular invasion and a younger age^9,10^. This data suggests that FAK inhibition is therefore a potential target in this aggressive phenotype, where limited treatment options are available. As such we further validated these findings in two triple negative PDX models. PDX are the most representative pre-clinical model producing faithful human breast cancer pathologies, unlike cell line models, with epithelial tumour heterogeneity and stromal tissue support. Thus, providing a complex human tumour environment to evaluate effects of FAK inhibition in combination with Paclitaxel on classic tumour phenotypes, e.g., tumour size and proliferation. As well as using the gold standard limiting dilution assay which allows tumour initiation, the ultimate CSC characteristic, to be measured after in vivo tumour treatments^32^, rather than the isolated CSC properties within our in vitro CSC assays.

In two triple negative PDX models, we demonstrated combined FAK inhibition and Paclitaxel treatment consistently reduced tumour growth, proliferation and CSC activity. In vivo FAK inhibition led to a reduction in mammosphere formation and ALDH^+^ expression ex-vivo, which was not seen consistently after Paclitaxel treatment alone. Combined VS4718 and Paclitaxel treatment also prevented the enrichment of ALDH+ expression seen in RC37 PDX model after Paclitaxel monotherapy. Previous combination studies using FAK inhibitor VS6063 and Paclitaxel has been shown to overcome taxane resistance in an ovarian cancer xenograft model, resulting in >90% reduction in tumour growth^16^. However, we demonstrate that combined FAK inhibition and Paclitaxel reduces tumour initiating capacity and subsequent tumour re-growth in patient derived samples, (PDX) in line with MDA-MB-231 and SUM159 xenograft studies^12^. Clinical use of a FAK inhibitor in triple negative breast cancer is likely to be given in combination with the current standard regime of chemotherapy, given the poor outcome associated with this phenotype. We believe this data demonstrates the potential additional benefits of combining FAK inhibition with existing adjuvant therapies to reduce resistant, residual disease which is enriched for CSCs.

Despite being the most representative pre-clinical model PDX models are grown in severely immunodeficient mice, and thus the role of FAK in regulating immune cells and the tumour micro-environment (TME) could not be investigated in this work. FAK inhibition in a pancreatic ductal carcinoma (PDAC) model promotes CD8+ cytotoxic T cell recruitment and creates a less fibrotic TME, rendering previously resistant PDAC cells sensitive to Gemcitabine, T cell therapy and PD1 agonists^29^. The dense and diffuse stroma, desmoplasia, which dominates the PDAC TME plays a major role in driving treatment resistance in pancreatic cancer and although less prominent in breast cancer it would be interesting to correlate CD8+ and FAK expression in a breast cancer cohort. Evidence of dual actions of a FAK inhibitor directly on tumour CSCs and the TME would be an advantageous. Recent studies in mouse mammary tumour models show when FAK inhibitor is used in combination with endogenous or exogenous signals that promote activation of T-cell co-stimulatory pathways, e.g., CD80, 4-144BB, and Ox40, anti-tumour immune responses are elevated and were capable of regression in some mouse models^33^.

Effects of FAK inhibition on metastasis could not be monitored within our triple negative PDX models as spontaneous metastasis were not present in the timeframe of tumours reaching their maximum tumour burden. FAK deletion has been shown to reduce lung metastasis in murine models^12^ and FAK inhibition with VS4718 reduced metastasis in both 4T1, CAL-51, and MDA-MB-231 xenograft models^12,30^. Therefore, investigation with an appropriate metastatic PDX model would be warranted as CSCs have been suggested to initiate breast cancer metastasis, as well as FAK inhibition switching off classical focal adhesion driven cell migration.

One key issue with targeting CSC in clinical trials is the lack of validated biomarkers of CSC activity/response, which can be easily measured in the lab using mammospheres, ALDH^+^ expression and the gold standard limiting dilution model, measuring in vivo tumour initiaiton^32^. We utilised ALDH1 and ITGα6, previously shown to be two of the better markers in breast cancer patient tissue^23,31^ however, these markers do not translate to PDX models. Ideally a hetereogenous patient population would be utilised in the form of a pre-surgical window or neoadjuvant trial to identify and validate biomarkers in early breast cancer. This study should include pre-treatment and post-treatment biopsies measuring FAK, CSC markers, and other pathways shown to be involved in CSC activity such as β-Catenin, alongside established laboratory based functional assays of CSC activity to further evaluate the response to FAK inhibition.

FAK inhibition alone reduces CSC activity across all molecular phenotypes in patient samples. High FAK expression is associated with poor clinical outcome and co-expression of high FAK and CSC markers identify a high risk patient population, who may benefit from FAK inhibition. Combined FAK and Paclitaxel treatment consistently reduced CSC activity in cell lines, patient samples and two triple negative PDX models. We believe this data provides evidence that the effects of FAK inhibition in addition to chemotherapy should be evaluated further in a neoadjuvant trial in early breast cancer.

## Methods

### Invasive ductal carcinoma cohort

A one recurrence (case) to two non-recurrence (control) cohort of 244 patients with invasive ductal carcinoma of non-specific type was created using formalin fixed paraffin embedded cancer samples. We obtained ethical approval from the Health Research Authority for this work under project code REC14/SW/1170. These patients underwent surgical resection between 2008 and 2011 at University Hospital South Manchester, with a median follow-up time of 75 months. Electronic and paper records were then used to record the patients clinical and histological details. The clinicopathological characteristics of this cohort are described in Supplementary Table 1. Tissue Micro Arrays were constructed with two epithelial cores and one stromal core taken from each sample under the guidance of Angela Cramer.

### Immunohistochemical staining

All tissue was processed and cut in the same way by the histology department at the Manchester Cancer Research Centre. Four millimeter sections were mounted onto slides and baked at 60 °C overnight. Once baked these slides were stained on the Leica Bond Max (up to a maximum of 30 slides per run). During this automated process sections were deparaffinised and dewaxed prior to heat-induced antigen retrieval. For each antibody we ran a positive control and an isotype negative control to ensure antibody specificity. Once stained, slides were dehydrated in a series of alcohols (70, 90, and 100%) for 3 min each and then placed in xylene for 5 min. Cover slips were added after being mounted with permount solution.

### FAK staining

Immunohistochemical staining for phospho FAK and total FAK was performed using the Leica bond (Leica microsystems, Wetzlar, Germany). Four micrometer paraffin embedded sections were cut 24 h prior to staining and baked at 60 °C overnight. Staining for phosphorylated FAK was undertaken using 44-624G Invitrogen antibody that binds to phosphorylation site Y397. IHC had already been optimised by the breast biology group at a concentration of 0.5 µg/ml, using epitope retrieval solution 2 (pH9) for 20 min, protocol F, casein blocking agent and the refine polymer detection kit. pFAK was scored via visual microscopy of TMA staining and high expression was defined as 2 or 3+ intensity in 90% of cells.

Immunohistochemical staining for tFAK was undertaken on the Leica bond using anti-FAK clone 4.47, Merck Millipore at a 1 in 250 dilution. Epitope retrieval solution 2 (pH 9) was used for 20 min using standard protocol F and the refine polymer detection kit. tFAK was scored via visual microscopy of TMA staining and high expression was defined as 3+ intensity in 20% of cells.

### ITGa6 staining

Immunohistochemical staining for ITGα6 was undertaken on the Leica bond using Atlas antibody HPA012696. We used a 1 in 250 dilution, epitope retrieval solution 2 (pH 9) for 20 minutes, standard protocol F and the refine polymer detection kit. ITGα6 staining was scored via visual microscopy of TMA staining and high expression defined using Friedrich’s et al criteria of grade III and IV expression in greater than 5% of epithelial cells.

pFAK, tFAK, and ITGα6 were scored using visual microscopy by two independent scorers, Simon Timbrell and Hosam Aglan. These scorers received training from Angela Cramer who counter scored 10% of samples and checked any discrepancies between the two scorers. Tissue samples were scored based on intensity and the percentage of epithelium staining positive with 500 cells per core being counted.

### Dual ALDH1/CK18 immunofluorescence staining

Given the non-specific nature of the IHC ALDH1 staining, we utilised an immunofluorescent protocol with co-staining using epithelial marker Cytokeratin 18 staining. We used the BD bioscience ALDH1 antibody 611195 at a 1 in 250 dilution. For the CK18 staining we used the Ab668 antibody at a 1 in 2000 dilution.

Slides were prepared as described above and then loaded on the Ventana DISCOVERY Ultra automated system. Following deparaffinisation slides were incubated for 8 min with cell conditioner 2 solution (pH6 solution). DISCOVERY Inhibitor was added for 8 min to inactivate endogenous tissue enzymes. Ab668 (ALDH1 antibody) was manually applied at a 1 in 250 dilution and incubated for 60 min. Slides were then incubated with DISCOVERY Omni Map anti-Ms HRP (horseradish peroxidase-linked anti-mouse IgG) for 16 min. The red fluorophore Opal670 (1 in 100) was then manually applied to slides to visualise ALDH1 staining and incubated for 12 min. The above sequence was repeated for the CK18 antibody (1 in 2000). The green Opal540 (1 in 100) fluorophore was used to visualise CK18 staining. Slides were washed twice in EZ Prep solution for 5 min and then in water once, for 5 minutes. Slides were mounted with Prolong Gold which contains a DAPI nuclear stain. Liver tissue was used as a positive control for ALDH1 staining. Dual staining was scored via automated detection using the HALO analysis software. With high staining being defined as >5% positivity within epithelial cells.

### Ki67 staining

Immunohistochemical staining for Ki67 was undertaken on the Leica bond using DAKO antibody (M7240) at a 1 in 250 dilution with epitope retrieval solution 2 (pH 9) for 20 min, standard protocol F and the refine polymer detection kit. Once stained these sections were scanned using the Leica scanner and the presence of DAB staining evaluated using the HALO analysis software.

### Cell lines

MCF10a and DCIS.com lines were purchased from Asterand. MCF7, BT474, SKBr3, MDA-MB231, and SUM159 lines were purchased from ATCC. Human breast carcinoma cell lines were grown in monolayer in their corresponding media and maintained in a humidified incubator at 37 °C at an atmospheric pressure of 5% (v/v) carbon dioxide/air. Cells were passaged via washing with PBS, addition of trypsin for 2–4 min and then spun down into a cell pellet. The supernatant was discarded, and the cells re-suspended in media and placed in a new flask.

### Mammosphere culture

Prior to the isolation of cells, 6-well plates were pre-coated with 1 ml of polyhema and baked for 48 h at 60°C. Single cells were isolated from cell lines, primary or in vivo tissue. In order to ensure single cell suspension, further disaggregation was undertaking by passing the cell solution through a 25G needle a maximum of three times at which point the cell concentration would be re-counted.

These single cells were seeded in mammosphere media at densities of 500 cells per cm^2^ and incubated at an atmospheric pressure of 5% (v/v) carbon dioxide/air. Cell lines were incubated for 5 days whilst human samples and PDX tumours were incubated for 7 days prior to counting. After incubation mammospheres were counted based on a size greater than 50 mM at 40 times magnification. The mammosphere forming efficiency was calculated as shown below:

$$\begin{array}{l}{\mathrm{Mammosphere}}\,{\mathrm{forming}}\,{\mathrm{efficiency}}\,\left( {{\mathrm{MFE}}} \right) \\= \frac{{{\mathrm{Number}}\,{\mathrm{of}}\,{\mathrm{mammospheres}} \, > \, 50{\mathrm{\mu M}}}}{{{\mathrm{Number}}\,{\mathrm{of}}\,{\mathrm{single}}\,{\mathrm{cells}}\,{\mathrm{seeded}}}} \times 100{\mathrm{ }}\end{array}$$

In order to evaluate CSC self-renewal, these primary mammospheres were then passaged without further treatment into non-adherent culture conditions. Primary mammospheres were counted and then extracted from the 6-well plates into a universal container and centrifuged at 200×*g* for 2 min. Mammospheres were then dissociated into single cells via enzymatic digestion with trypsin and mechanically disaggregated by passing through a 25G needle for ten times. Single cells were re-seeded in mammosphere culture conditions described above with no further treatment. After 5 days of incubation (7 days for primary cells) the number of secondary mammospheres formed were counted and the following calculation used to evaluate mammosphere self-renewal:

$${\mathrm{Mammosphere}}\,{\mathrm{self}}\,{\mathrm{renewal}}\left( {\% {\mathrm{MS}}} \right) = \frac{{2^\circ {\mathrm{mammospheres}}\left( { > 50{\mathrm{\mu M}}} \right)}}{{1^\circ {\mathrm{mammospheres}}}} \times 100.$$

Many common materials and methods were as described Williams et al.^14^ and the mammosphere protocol described in Shaw et al.^21^. IHC antibodies are as follows; FAK, anti-FAK clone 4.47, Millipore, 1:250 dilution; PTyr397 FAK, 44-624 S, Invitrogen, 0.5 µg/ml; ITGα6, HPA012696, Atlas, 1:250; Ki67, M7240, DAKO antibody,1:250. All blots derive from the same experiment and were processed in parallel.

### Immunoblot analysis

Lysates were collected as outlined in this section unless otherwise stated. Cells were grown in monolayer in 10 cm^2^ petri dishes for 24 h. Cells were then washed twice with ice cold PBS and 250 µl of lysis buffer added to the dishes. After 2 min cells were removed via scraping. The detachment of cells was confirmed on microscopy. The cell lysate was the placed on a rotator at 4 °C for 1 h. After which the cell suspension was centrifuged for 10 min at 200 × *g* and the supernatant collected.

The BIORAD assay solution was then used to determine the protein concentration. Reference protein concentrations were made as per the manufacturer’s guidelines using a BCA assay included in the kit. Two hundred microliter of protein analysis solution was added to 10 µl of 1 in 10 diluted lysate sample. Protein concentrations were then calculated using the 552 nm laser on the VersaMax ELISA microplate reader. The amount of lysate required to load 40 µg of protein per lane was then calculated. This volume of lysate was then mixed with 4× Laemmli sample buffer. Proteins were then denatured by heating the lysates to 90 °C for 10 min.

Forty microgram of denatured lysate was loaded to each well of a Mini-PROTEAN gel with a 10 µl of precision plus kaleidoscope prestained protein standard used as a reference ladder. Gels were run at 120–200 V until the 37 kDa approached the bottom of the gel usually taking 1 h at 150 V. A wet transfer was undertaken using the Hybond-C extra nitrocellulose membrane at 30 V overnight. After transfer Ponceau S solution was used to assess the validity of protein transfer.

Membranes were blocked with their corresponding blocking agents shown in Table 2.1.7 for 1 h and then washed three times. Each was undertaken with TBS-Tween for 10 min. Primary antibodies were made up in the same solution that the membrane was blocked with and incubated at room temperature for 1 h or overnight at 4 °C. Membranes were washed, re-blocked and exposed to the appropriate secondary HRP prior to ECL exposure. Membranes were developed using the BIORAD ChemiDoc touch system and relative protein densities analysed using Image Lab software.

### Aldefluor assays

The Aldefluor assay kit from STEMCELL Technologies was utilised to identify ALDH^+^ expression. 1,000,000 cells were suspended in 1 ml of aldefluor buffer to which 5 µl of BAAA was added and incubated for 40 min at 37 °C. Each sample had a DEAB control with a 0.1% gate utilised during the quantitative analysis. During the flow sorting analysis 7AAD (Bioscience) was utilised as a live-dead stain. H2KD antibodies (Biolegend) conjugated with pacific blue fluorophore were used to exclude mouse stromal cells during PDX experiments.

### siRNA transfection

A set of four individual pre-designed siRNA’s targeting PTK2 (the gene encoding for FAK) were purchased from Dharmacon alongside positive control GAPDH and scrambled negative control. A range of concentrations of siRNA (25–50 nM) were trialled, at a range of different time points (48–72 h), on a range of different cell densities 10,000, 50,000, and 100,000 cells per 10 cm^2^ plate for MDA-MB 231 and SUM159 cells. siRNA constructs were used as single agents and in combination with the effect on tFAK expression measured by western blot analysis. Cells were seeded 24–48 h prior to transfection and washed prior to exposure to transfection media. The siRNA transfection media was prepared by adding 10 µl of siRNA and 190 µl of serum-free media to one tube and 10 µl of transfection reagent to 190 µl of serum free media in another tube. These separate tubes were incubated at room temperature for 5 min prior to mixing. Once mixed this solution was incubated for a further 20 min at room temperature prior to the addition of a further 1600 µl of DMEM media containing FCS and l-glutamine. This created a 50 nM solution in 2 ml of media, which was added to a 10 cm^2^ culture plate. Cells were incubated with this media for 48–96 h. After a maximum of 96 h cells were then collected for western blot analysis or isolated as single cells for mammosphere culture and Aldefluor expression analysis.

### Isolation of epithelial cells from human tumour samples

Primary patient tumours were collected by the Manchester Biobank on invasive ductal tumours >1.5 cm. Informed consent was taken by the Manchester Biobank team with a project ethics code of 05/Q1403/159. The MCRC Biobank is licensed by the Human Tissue Authority (licence number:30004) and has been ethically approved as research tissue bank by the South Manchester Research Ethics Committee (Ref:18/Nw/0092).

Excised surgical specimens were placed in RPMI media with FCS, l-glutamine, penicillin and streptomycin, and a piece of tumour removed under pathologist review. Tumours were cut into chunks and enzymatically digested using the Miltenyi tumour dissociation kit and protocol.

### Patient derived xenograft studies

All in vivo studies were carried out in accordance with UK home office (Scientific Procedures) Act 1986 under project licence PCFEC0D4 and with approval of the CRUK Manchester Institute Animal Welfare and Ethics Review Board. We selected two triple negative PDX models based on RNA expression of FAK (PTK2B), ALDH1A1 and ITGα6 expression, Supplementary Fig. 5b. FAK and pTyr397FAK IHC expression was confirmed as moderate/high within the RC37 and RC193 model selected (Supplementary Fig. 5c, d). Tumours were implanted fresh as 2 × 2 mm chunks into bilateral flanks of NOD scid gamma (NSG) female mice aged 6–8 weeks. Mice were measured twice weekly and all treatment was started at the same time point when combined tumour volume was 250–300 mm^3^ per mouse, with treatment lasting up to 4 weeks as shown in the overview in Supplementary Fig. 5a. Control mice received vehicle only. VS4718 was given via oral gavage at 50 mg/kg B.D on weekdays only. Paclitaxel was administered weekly via intraperitoneal injection 7.5 mg/kg. Tumour volumes were recorded twice weekly via calliper measurement until the combined tumour burden exceeded 800 mm^3^ at which point it was measured daily. All mice were checked for visible signs of toxicity and weighed daily; throughout the treatment no toxicity was seen. Tumours were harvested when total tumour burden per mouse reached 1250 mm^3^ and placed in media prior to processing.

In order to evaluate the effects of FAK inhibition and paclitaxel treatment on the tumour initiating capacity of treated mouse tumours, we undertook a limiting dilution assay. During this assay single cells from treated PDX tumours were injected subcutaneously into the flanks of 6–8-week-old NSG mice at a range of cell dilutions. Cells were defrosted, spun down and viable cells counted with trypan blue prior to injection. Each mouse received an injection made up of 200 ml per flank containing 100 ml of media and 100 ml of matrigel. This matrigel media mix contained a range of cell concentrations and was injected via 25G needle.

### Statistical analysis

Cancer disease and recurrence free survival were calculated using cox-regression (Hazard Ratio and 95% confidence intervals). In vitro mammosphere data are represented as mean ± SEM taken over ≥3 independent experiments, ≥3 technical replicates per experiment, unless otherwise stated. Statistical significance was measured using analysis of variance (ANOVA) test with post-hoc Tukeys or Dunnett’s multiple comparison, or student *t*-test using graph pad prism. A *p* value of less than 0.05 was considered significant and all statistical tests were two-sided. All statistics were carried out under the guidance of statistician P Foden.

### Reporting summary

Further information on research design is available in the Nature Research Reporting Summary linked to this article.

## Supplementary information

Supplementary material

Reporting Summary

## Data Availability

The data generated and analysed during this study are described in the following data record: 10.6084/m9.figshare.14152460^34^. The following data are openly available as part of the data record: mammosphere counts and tumour growth plots in the Prism file “NPJ_data.pzfx”; Aldeluor FACS plots in the files “SiRNA_ALDH.wsp” and “RC37_ALDH.wsp”; IDC cohort data in the SPSS file “IDC_IHC.sav”. For tissue staining for tFAK, ITGa6, Ki67 and ALDH, the associated files are available upon reasonable request from the corresponding author.
