# Supplementary material for: FAK inhibition alone or in combination with adjuvant therapies reduces cancer stem cell activity
Source: NPJ Breast Cancer. 2021 May 28;7:65. doi: 10.1038/s41523-021-00263-3 (PMC8163772; doi:10.1038/s41523-021-00263-3)
Supplement: Supplementary file 1 — Supplementary material [file 41523_2021_263_MOESM1_ESM.pdf]

Supplementary table 1: Overview of the literature evaluating FAK expression in breast cancer.

Author	Method	Cohort details	High Expression Criteria	Prognostic outcome	Association	Comments
Almstedt 2017	IHC - FAK CST 1:100	German cohort. 335 lymph node negative patients.43 local recurrences, 75 distant metastases	Modified Allred ≥6_ (45.1%) 22.4% had 3+ intensity expression	Recurrence risk HR **1.54** (1.04- 2.28) Uni,p=0.03 **HR1.28 (**0.85- 1.92) Multi, p=0.244	Particularly prognostic in ER- /HER2+ve	Lymph node negative only Mainly ER+/HER2- (234)
Golubovskaya 2014	IHC –FAK 4.47 clone	US cohort 196 patients of whom 117 metastasised	>4 - Each core scored as 0-3 based on intensity and number of positive cores counted.	Reduced breast cancer survival. 44 months vs 123months (p=0.003)	Young Age LVI Triple Negative	High FAK in metastatic tissues is associated with reduced survival
Yom 2011	IHC –FAK 4.47 clone<br><br>FISH	South Korean 242 patients<br><br>393 patients	3+ intensity in<br><br>≥20% epithelial cells	Breast cancer death **HR 1.38** (0.7-2.72) p=0.36 **HR 3.62** (1.817.25) p=<0.001	Triple Negative, p53, Ki67 Correlates with IHC	FISH positivity is associated with reduced survival.
Alexopoulou 2014	IF - FAK 4.47 clone Conjugated 546nm	UK cohort 149 samples	Automated fluorescence analysis	No clinical follow up	Triple Negative High Grade ER negativity	
Lark 2005	IHC –FAK 4.47 clone 1:250	US cohort 629 patients	3+ or 4+ intensity in ≥ 90% of epithelial cells.	No clinical follow up	High grade. ER negative HER2 positive	
Schmitz 2004	IHC -FAK Santa Cruz 1:100	162 – node negative 19 breast cancer deaths	3+ intensity in ≥20% of epithelial cells	Not given	High grade ER negative HER2 positive	Associated with Akt 22.5% -3+
Lightfoot 2004	IHC - FAK 4.47 1:250	50 Benign 51 DCIS 18 IDC	3 or 4+ intensity ≥ 90% epithelium	No clinical follow up.	Higher expression in DCIS/IDC	
Williams 2015	IHC - pTyr397FAK 44-624G<br><br>FAK 4.47	63 DCIS samples 21 recurrences	≥ 90% 2 or 3+ intensity	Risk of recurrence HR **3.44** (1.189.31) p=0.015 FAK- No association with prognosis	Recurrence in DCIS.	DCIS only
Sheen-Chen 2013	IHC - pTyr397FAK ab4803 1:100	98 Recurrence number not stated	3+ intensity	Breast Cancer Death HR 1.3 p=0.474	Not associated with survival in IDC.	

IHC = Immunohistochemical analysis, CST = Cell Signalling Technologies, FAK= Total FAK, FISH = Fluorescent In Situ Hybridisation, IF – Immunofluorescence, pFAK = pTyr397FAK

Supplementary table 2: Clinical and pathological characteristics in IDC cohort.

Variable	Categories	Number (%)	Recurrence (n=89)	Non-Recurrence (n=155)	p-value
**Age (years)**	Median (IQR)	244	58 (47-69.5)	57 (49-67)	0.605*
**Tumour Size (mm)**	Median (IQR)	242	25 (16-38.5)	20 (13-34	0.132*
**Surgery**	WLE	**57.4%** (140)	**51.6%** (46)	**60.6% (**94)	0.150**
	Mastectomy	**42.6%** (104)	**48.3% (**43)	**39.4% (**61)	
**Lymph node**	Positive	**40.5%** 99	**57.3%** (51)	**31.3% (**48)	**<0.001****
	Negative	**58.6%** 143	**42.7%** (38)	**68.7% (**105)	
	Missing	**0.9%** (2)			
**Excision Margin**	Incomplete (<1mm)	**33.4% (**57)	**13.5%** (12)	**29%** (45)	**0.004****
	Complete (>1mm)	**76.6%** (187)	**86.5%** (77)	**71.0%** (110)	
**Tumour grade**	3	**66.7%** (162)	**70.8%** (63)	**63.9%** (99)	0.497**
	2	**25.8% (**63)	**23.6%** (21)	**27.1%** (42)	
	1	**7.8%** (19)	**5.6%** (5)	**9.0%** (14)	
**Multifocality**	Yes	**17.6%** (43)	**21.8% (**19)	**15.6%** (24)	0.199**
	No	**81.1%** (198)	**78.2%** (68)	**86.4% (**130)	
	Missing	**1.2%** (3)			
**Ki67 (%)**	Median (IQR)	162	38 (25.4-66.5)	28 (12.5-46)	**0.025***
**Metastasis**	Yes	**23.4%** (57)	**64.0%** (57)	**0%** (0)	**<0.001***
	No	**76.7% (**157)	**36.0% (**32)	**100%** (155)	
**Breast Cancer Deaths**	Yes	**13.9%** (34)	**38.2%** (34)	**0%** (0)	**<0.001***
	No	**86.1%** (210)	**61.8%** (55)	**100%** (155)	

Continuous variables were compared using the independent samples t-test* and categorical data was analysed using the Pearson Chi square test**.

Supplementary Figure 1: Isolation of ALDH+ epithelial cells and ALDH sorting gates.

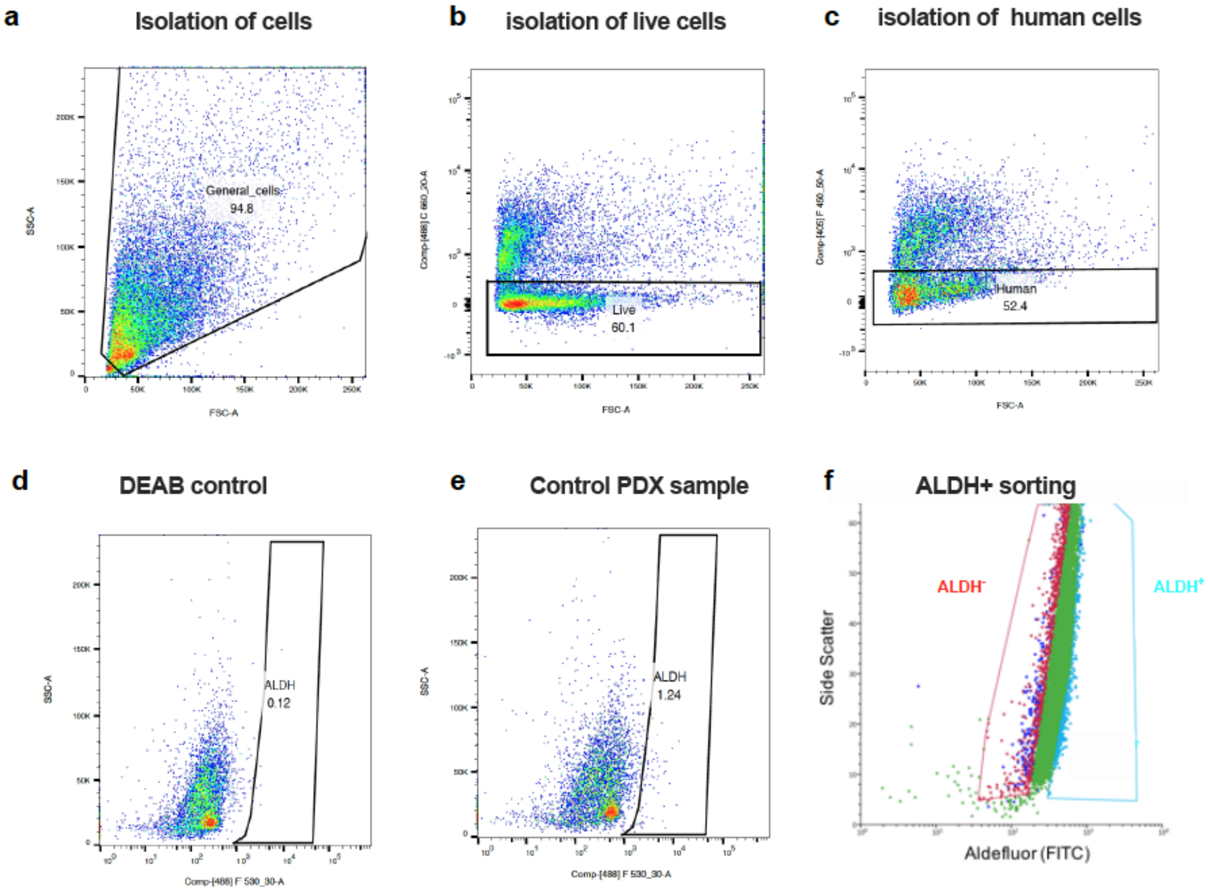

FACS plots demonstrating the identification of epithelial staining of ALDH was identified in PDX models and flow sort experiment. **(a)** General cells were selected, then **(b)** live cells isolated using 7AAD. **(c)** Mouse cells were then excluded using H2KD. For the Aldefluor assay the **(d)** DEAB control was set at 0.1% with an illustrative FACS plot of ALDH+ expression in mouse receiving control only. Shown in **(e)**. **(f)** Flow cytometry plot demonstrating the gating used to isolate the ALDH+ and ALDH- populations. ALDH+ was selected against a 0.1% DEAB control and the bottom 20% of ALDH expression identified as ALDH- expression.

Supplementary Figure 2: Pharmacological FAK inhibition and SiRNA knockdown reduces CSC activity in SUM159 cells.

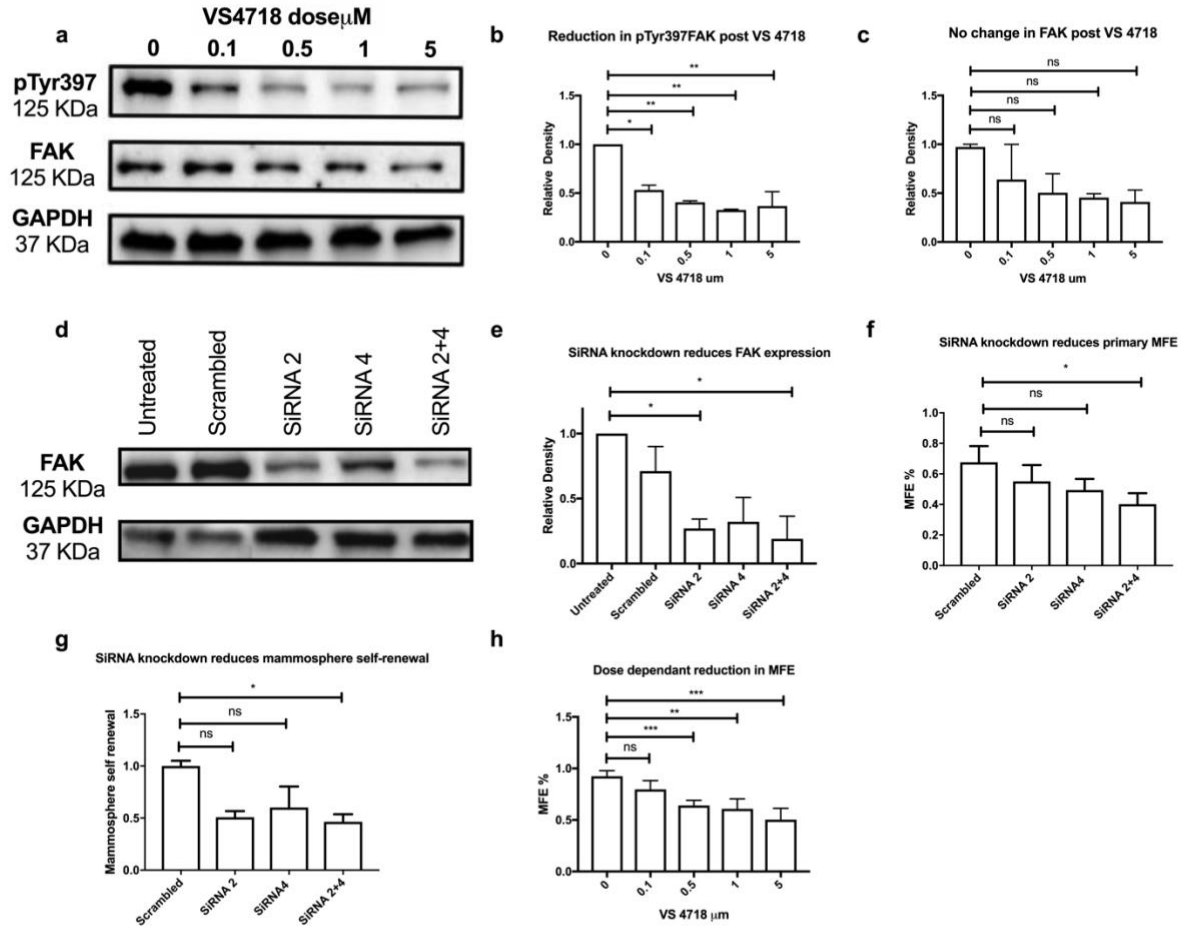

(a) Representative western blot demonstrating pharmacological FAK inhibition with VS4718 resulted in a dose dependent reduction in pTyr397FAK with relative protein expression shown in **(b)** but not **(c)** FAK (n=2). FAK knockdown using SiRNA resulted in a decrease in FAK expression as shown in the **(d)** western blot with **(e)** relative density plot. This reduction in FAK correlated with a reduction in **(f)** primary and **(g)** secondary sphere formation. **(h)** The dose dependent reduction in pTyr397FAK corresponded with a reduction in primary MFE. One-way ANOVA with post hoc Dunnett's test. (ns= not significant, *p<0.05, **p<0.01, ***p<0.001, ***p<0.0001). All data n=3 unless otherwise stated and error bars are mean ± SEM.

Supplementary Figure 3: High FAK is associated with reduced breast cancer survival, increased risk of metastasis and recurrence.

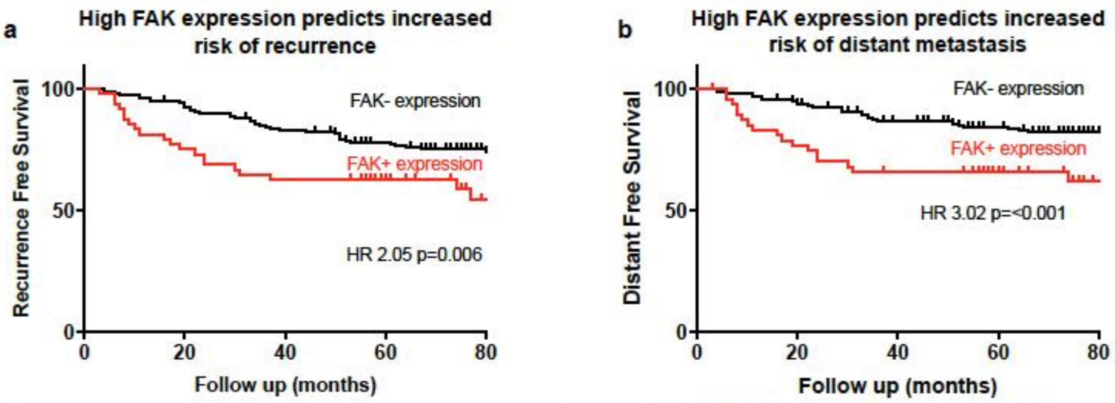

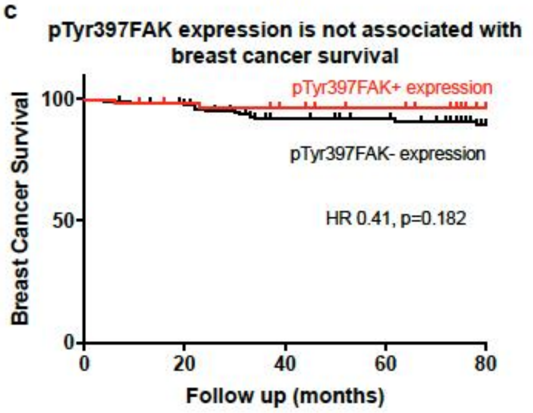

d

		pTyr397FAK Negative	pTyr397FAK Positive	p value
tFAK	Negative	**69.9%** (109/156)	**30.1%** (47/156)	
	Positive	**65.4%** (17/26)	**34.6%** (9/26)	0.646

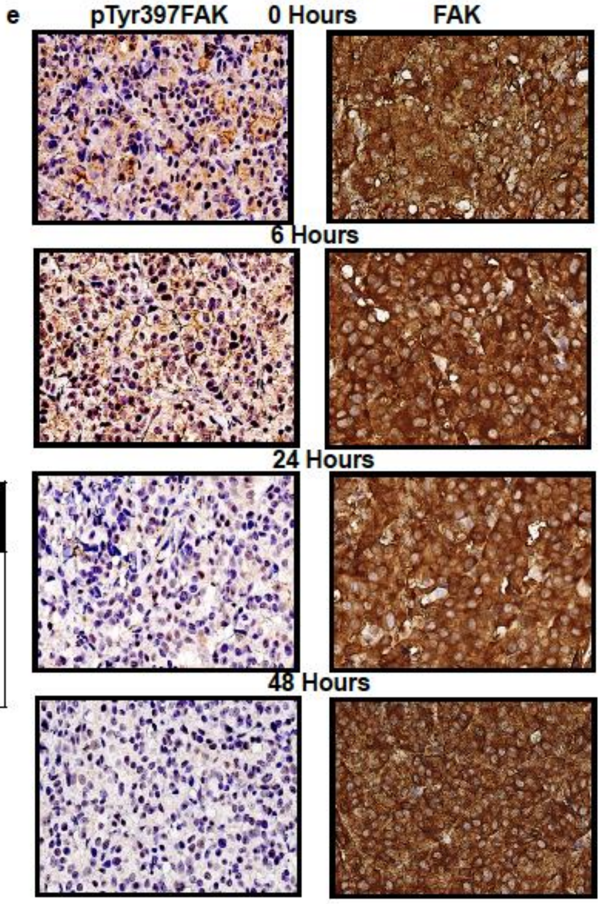

(a) Kaplan-Meier demonstrating that high FAK is associated with an increased risk of recurrence HR 2.05 (1.23 -3.43, Cox-proportional regression p=0.006). **(b)** Kaplan-Meier demonstrating that high FAK is associated with an increased risk of metastasis HR 3.02 (1.65-5.49, Cox-proportional regression p = <0.001). **(c)** Kaplan-Meier demonstrating epithelial pTyr397FAK does not predict breast cancer survival in our cohort. HR 0.41 (0.12-1.51, Cox proportional regression p=0.182). **(d)** Further analysis demonstrating no association between FAK and pTyr397FAK IHC staining with a

Kappa agreement of 0.03 and p value of 0.646. Tumours harvested from a PDX with high pTyr397FAK were then placed in media for 0, 6, 24 and 48 hours prior to fixation and processing. Photomicrographs taken at 40 x magnification demonstrate that **(e)** pTyr397FAK expression is lost with increasing time to fixation in the first column whilst the second column demonstrates that FAK expression does not change with increasing time to fixation.

Supplementary Figure 4: High ALDH1 and ITGα6 is associated with poor clinical outcome.

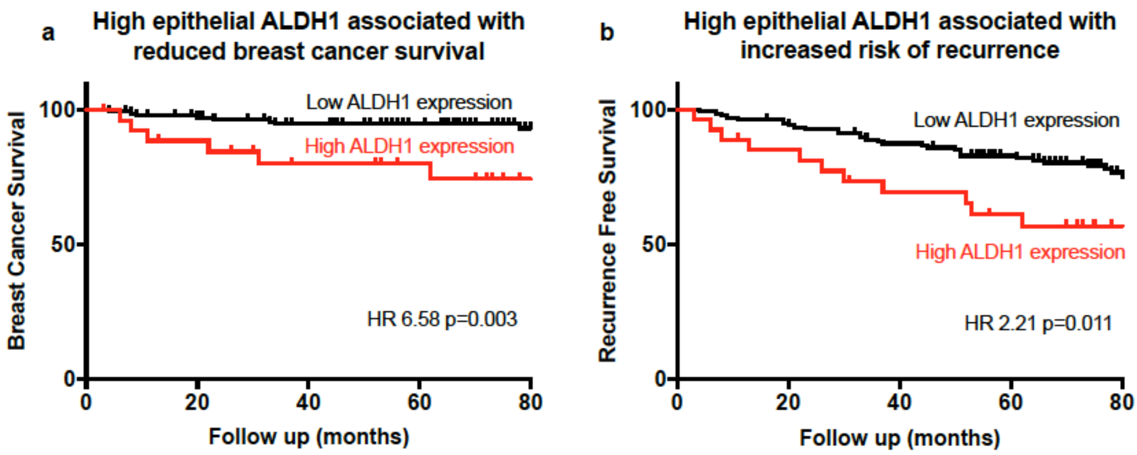

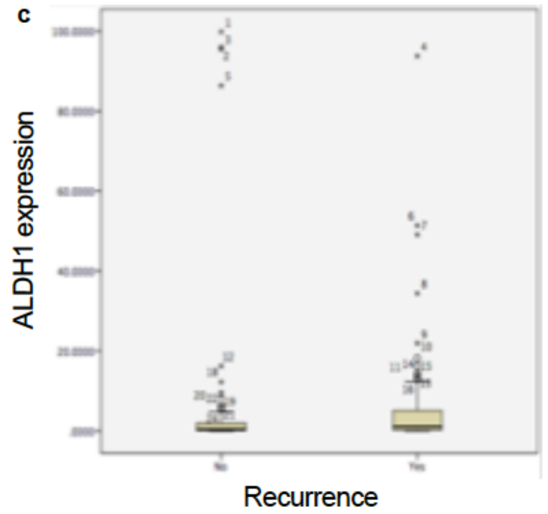

d

	Low ITGα6	High ITGα6	P value
Luminal A	**72.9%** (43/59)	**27.1%** (16/48)	
Luminal B	**75.7%** (50/66)	**24.3%** (16/66)	
HER2	**55.5%** (25/45)	**45.5%** (20/45)	<0.001
Triple Negative	**35.3%** (24/68)	**64.7%** (44/68)	<0.001

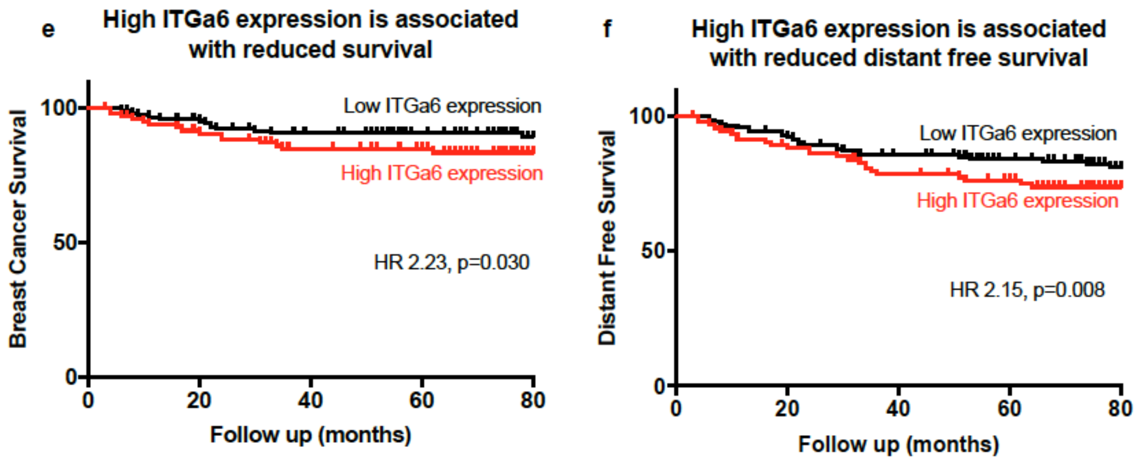

(a) Kaplan-Meier demonstrating that high epithelial ALDH1 expression is associated with poorer survival (n=165) HR 6.58, 1.87-23.10, p=0.003, cox-proportional hazard regression). **(b)** Kaplan-Meier demonstrating that high epithelial ALDH1 is associated with an increased risk of recurrence HR 2.21 (1.20-4.05, cox-proportional hazard regression, p=0.011). **(c)** Whisker plot demonstrating that any

epithelial ALDH1 expression is associated with an increased risk of recurrence. Average ALDH1 expression is 6.86% in recurrence samples as opposed to 4.99% in non-recurrence cohort (p=0.023, Mann-Whitney U test). **(d)** High ITGα6 expression is associated with a triple negative phenotype. Chi square test used to evaluate expression between categorical variables. **(e)** Kaplan-Meier demonstrating that high ITGα6 expression is associated with reduced breast cancer survival in our IDC cohort (n=232) HR 2.23 (1.08-4.58, cox-proportional regression, p=0.030). **(f)** Kaplan-Meier demonstrating that high ITGα6 expression is associated with increased rate of metastasis. HR 2.16 (1.22-3.81, cox-proportional regression, p=0.008).

Supplementary Figure 5: An overview of PDX experiments

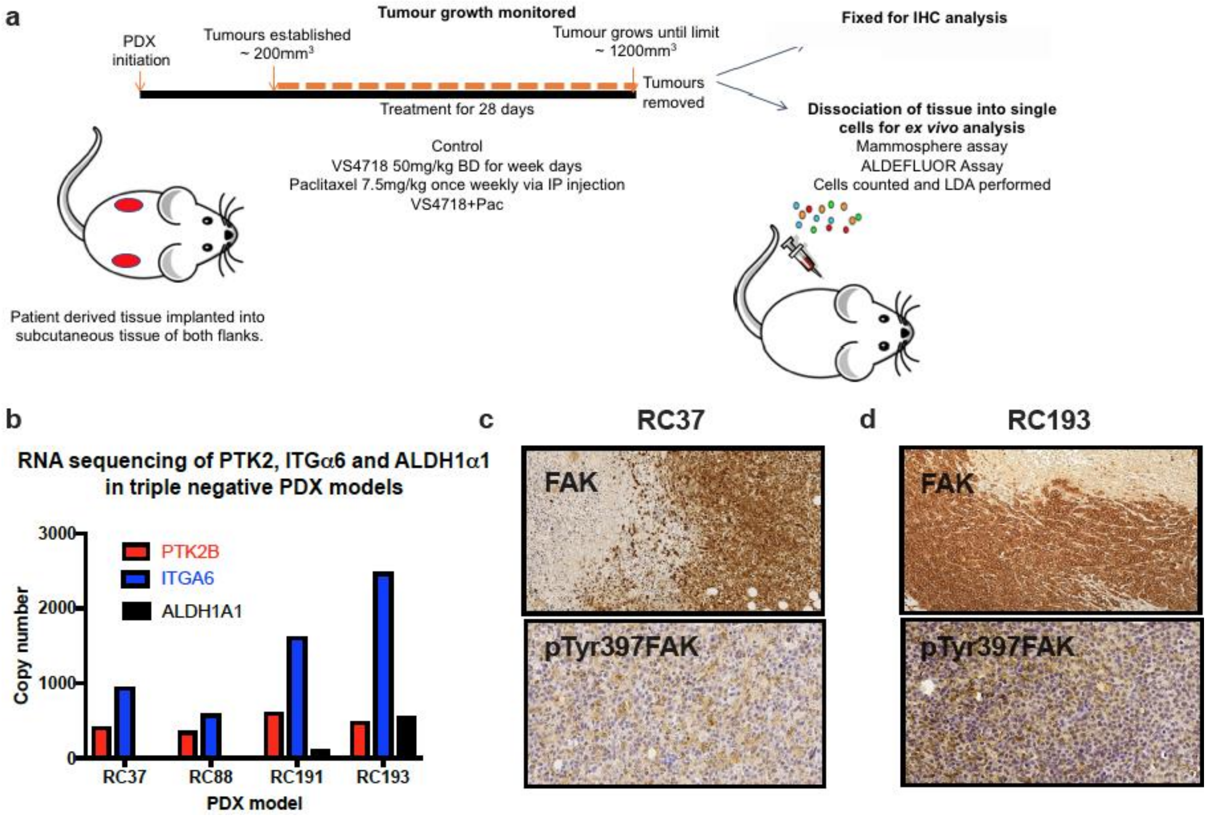

(a) Figure demonstrating how the PDX experiments were performed whereby chunks of fresh tissue from RC37 and RC193 are implanted into both flanks and treated for a maximum of 4 weeks whilst tumours are measured twice weekly and the daily when over 800mm^3. In the control group mice received vehicle control via oral gavage and DMSO+PBS IP injections once weekly. In the chemotherapy only group 7.5mg/kg Paclitaxel was given via IP injection once weekly. In the FAK inhibitor only group VS4718 via oral gavage twice daily on week days. In the combined group mice received both the above treatments. **(b)** Relative RNA expression of PTK2 gene encoding for FAK, ITGα6 and ALDH1A1 in the triple negative PDX models available. Photomicrographs demonstrating that **(c)** RC37 and **(d)** 193 express moderate to high FAK. Top image of FAK taken at 10 x magnification and bottom of pTyr397 staining taken at 40 x magnification.

Western blots included in Manuscript.

Figure 1a) pFAK CST 8556

Ladder (kda) MCF10a DCIS.comMCF7 BT474 SKBr3 MDA-MB231 SUM159 Ladder

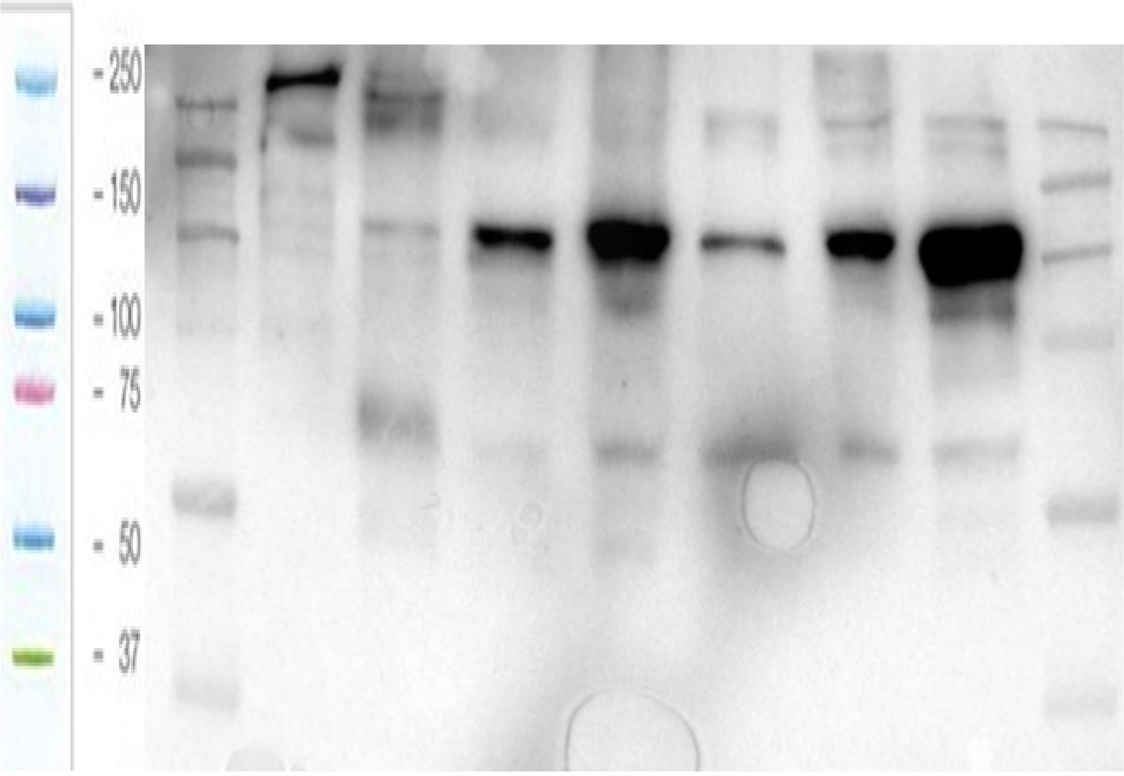

tFAK

Ladder MCF10a DCIS.comMCF7 BT474 SKBr3 MDA-MB231 SUM159 Ladder

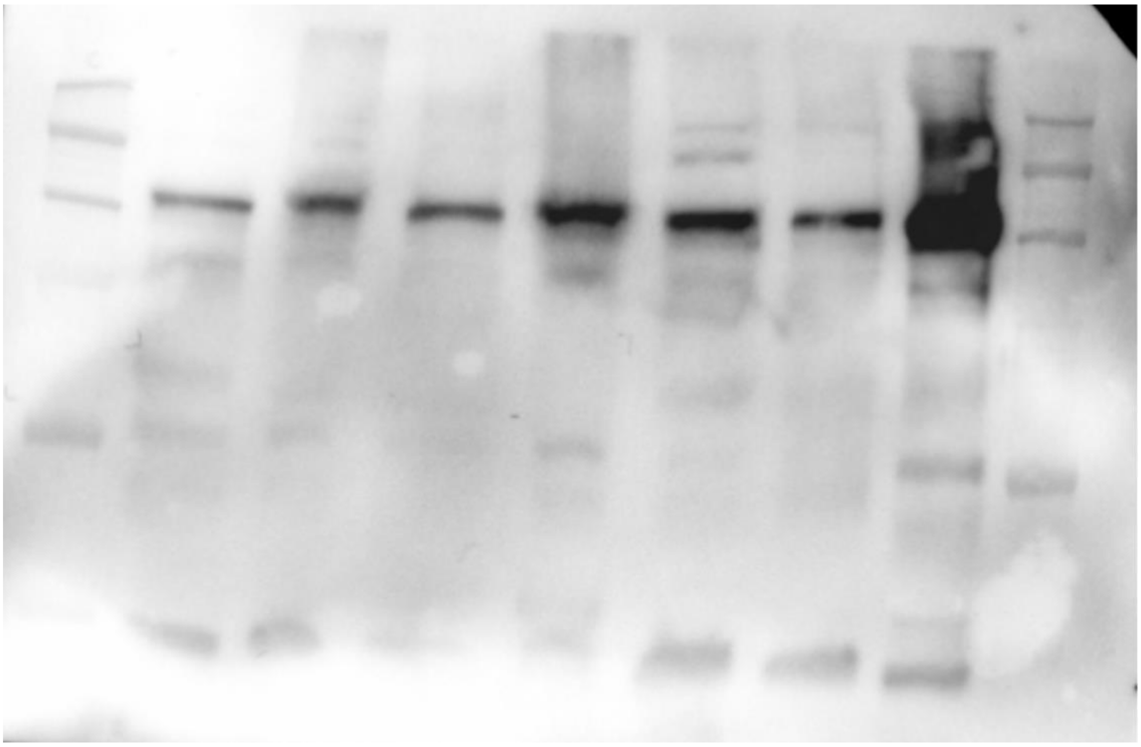

GAPDH

Ladder MCF10 DCIS.comMCF7 BT474 SKBr3 MDA-MB231 SUM159 Ladder

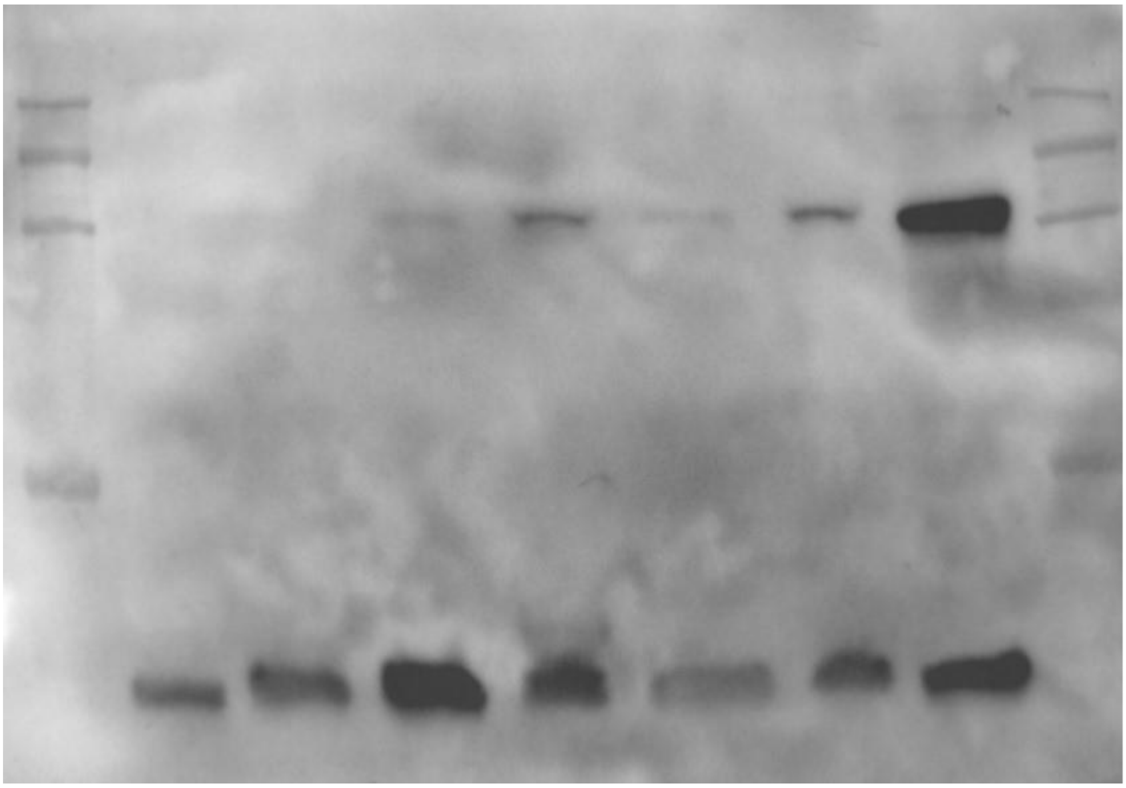

Figure 1c – ALDH flow sort

ALDH- ALDH+ Ladder

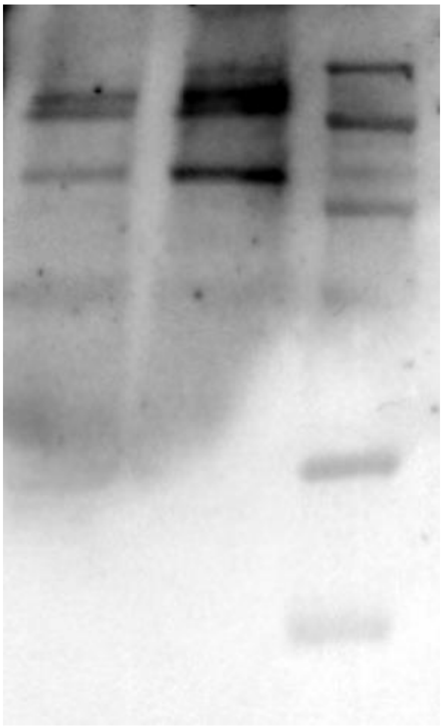

TFAK

ALDH- ALDH+ Ladder

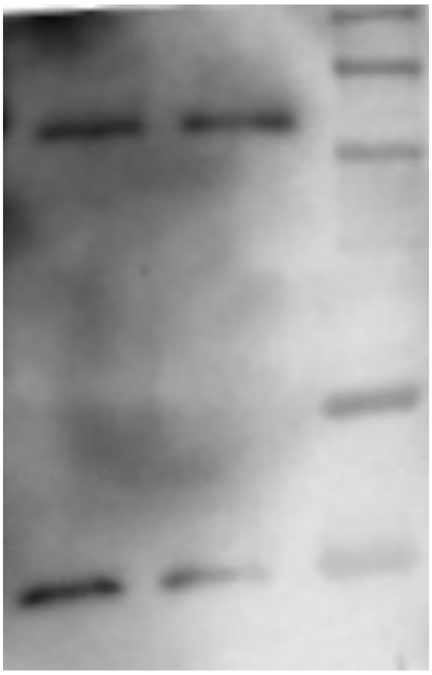

GAPDH
ALDH- ALDH+ Ladder

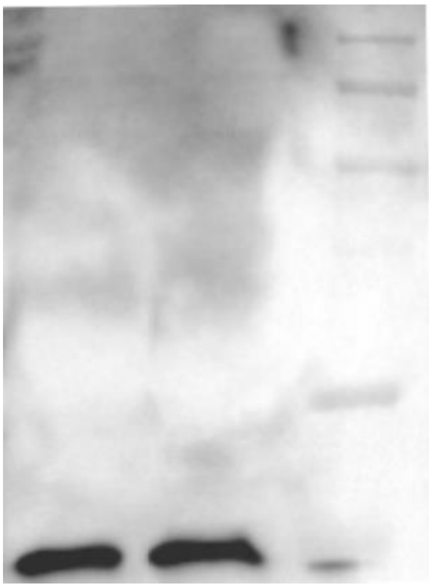

2a Time post 0.5μM Fig Figure 2A MDA MB 231 time course
VS4718 (hours) – pFAK CST 8556

Ladder 0 1 3 6 24

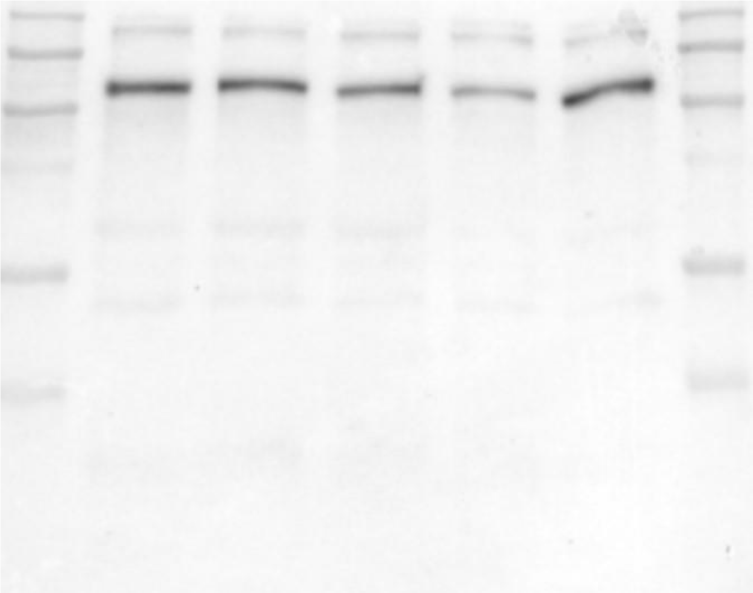

TFAK

Ladder 0 1 3 6 24

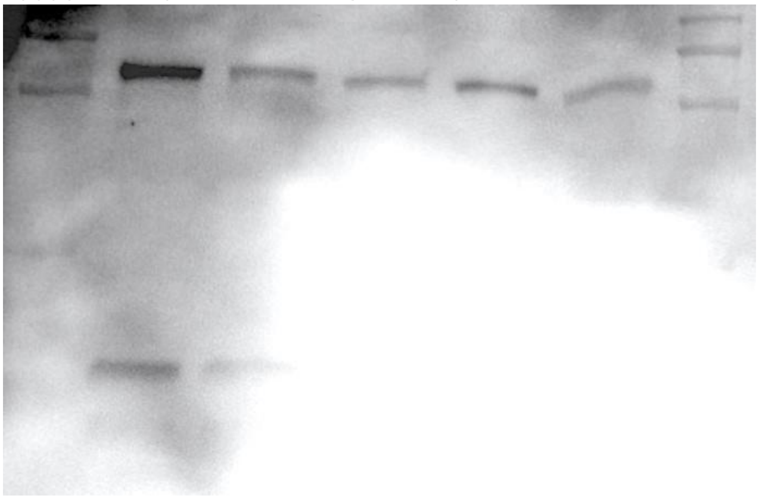

GAPDH

Ladder 0 1 3 6 24

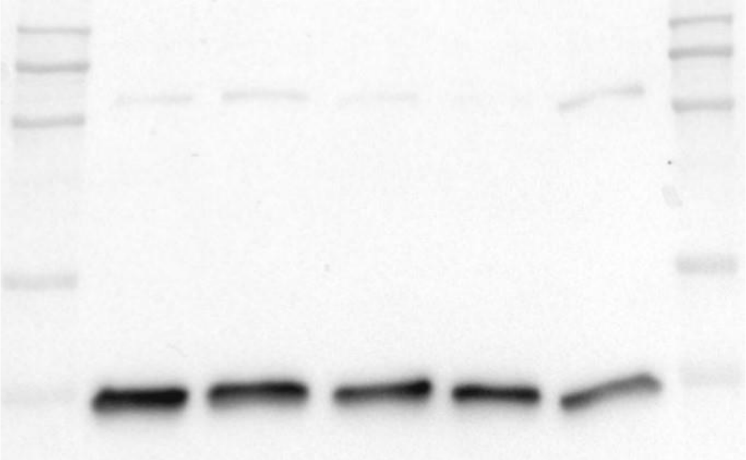

2C Dose response MDA-MB-231 cells

pFAK

Ladder 0 0.1 0.5 1 5

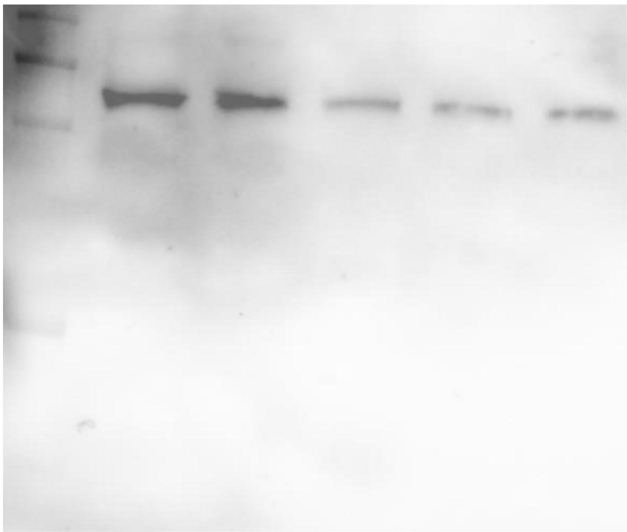

tFAK

Ladder 0 0.1 0.5 1 5

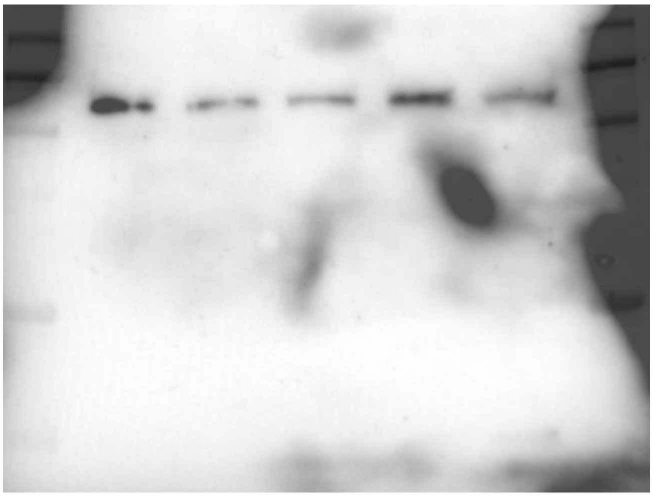

GAPDH

Ladder 0 0.1 0.5 1 5

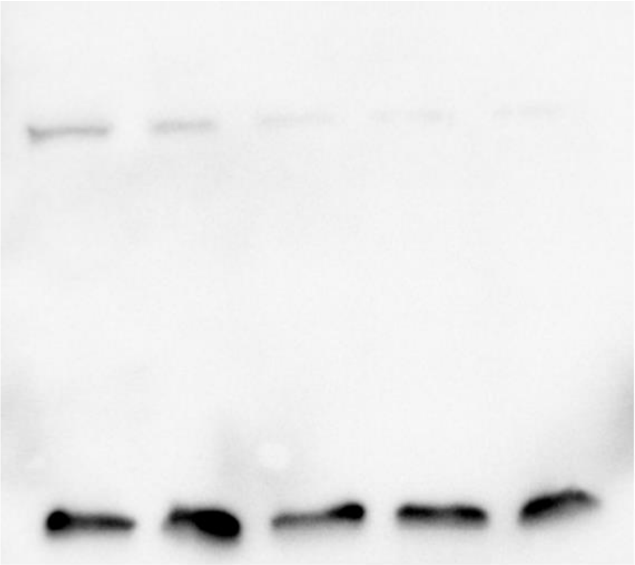

Unfortunately GAPDH doesn't have ladder but does appear to have remnants of pFAK staining above which corresponds.

2g SiRNA MDA-MB-231
tFAK
Ladder Untreated GAPDH Scrambled Si2 Si4 Si2+4

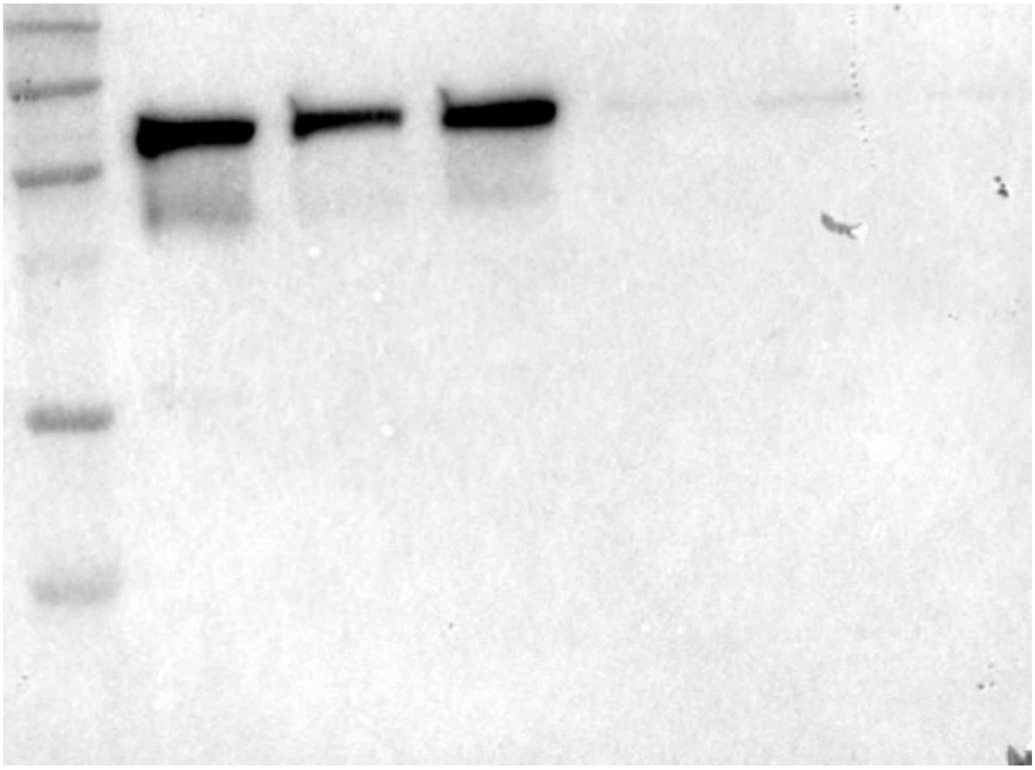

GAPDH
Ladder Untreated GAPDH Scrambled Si2 Si4 Si2+4

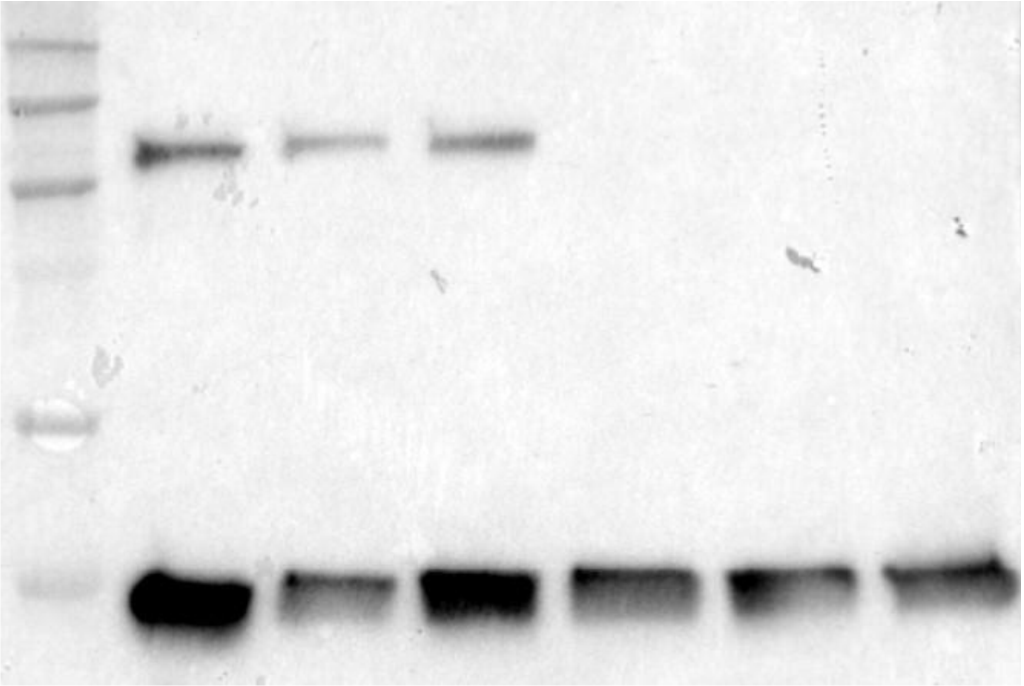

Supplementary figure 2a.
Post VS4718 dose – pFAK in SUM159
Ladder 0 0.1 0.5 1 5

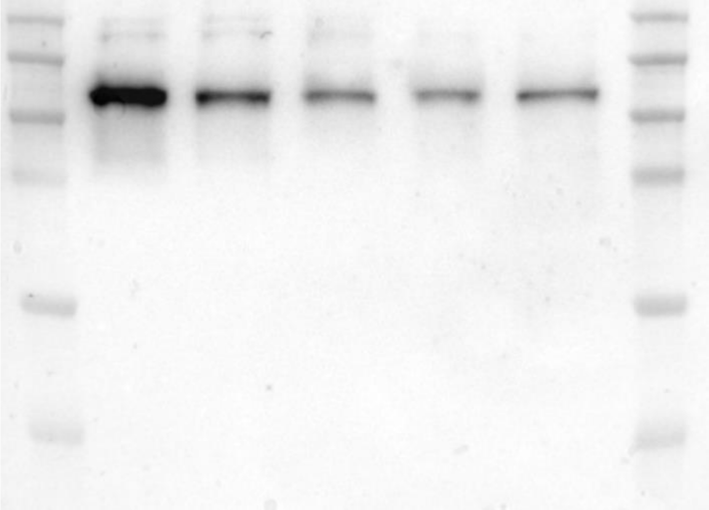

TFAK
Ladder 0 0.1 0.5 1 5

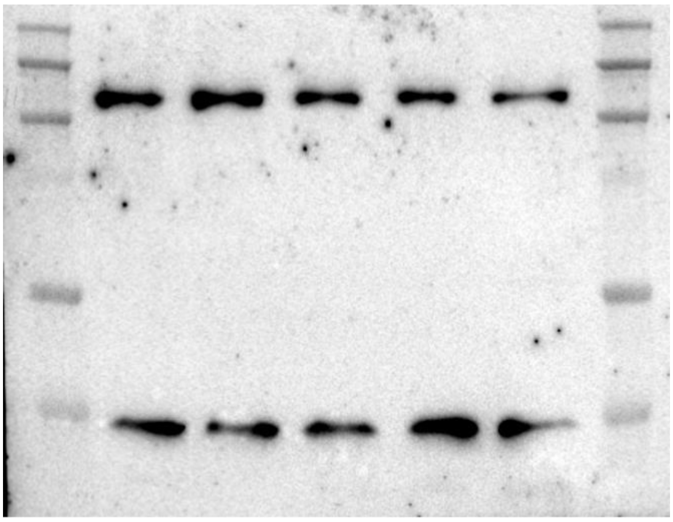

GAPDH – no ladder but it is there above where there is also some GAPDH stain.
0 0.1 0.5 1 5

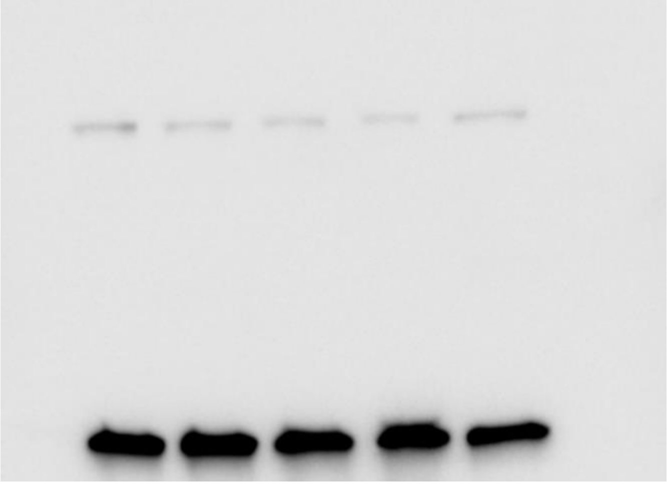

Supplementary figure 2d.
SUM159 – siRNA tFAK
Ladder Unrx Scr GAPDH Si2 Si 4 Si 2+4

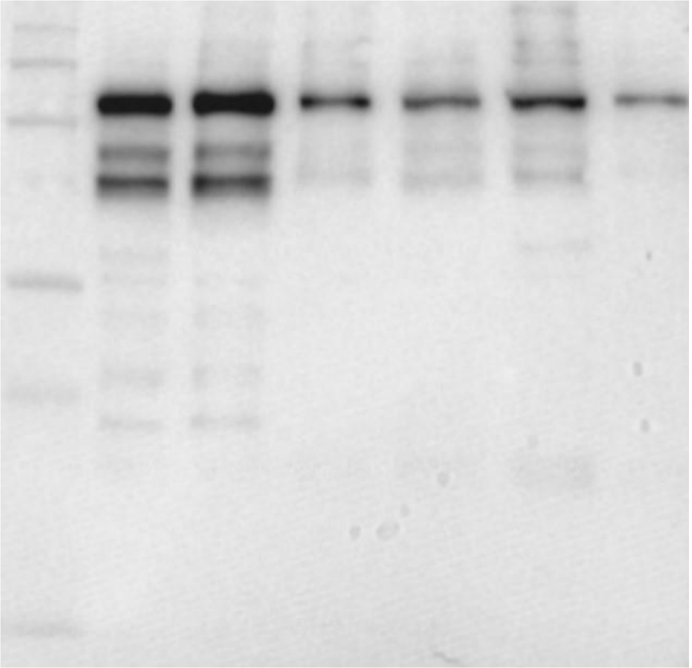

GAPDH
Ladder Unrx Scr GAPDH Si2 Si 4 Si 2+4

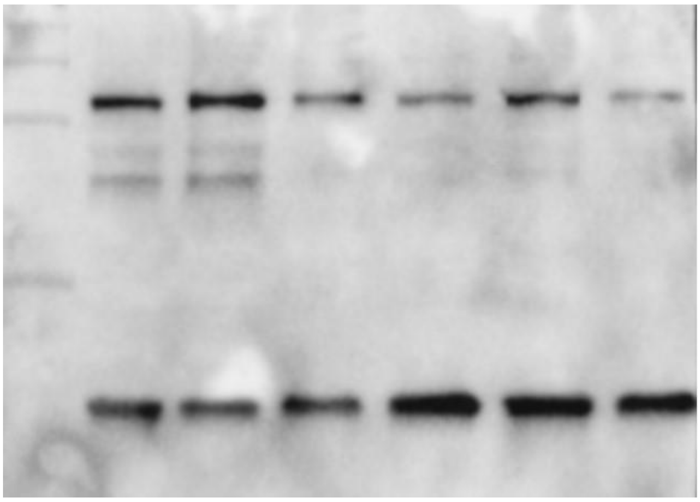

Note GAPDH column (3) wasn't in paper but can add. However, can't do comparative density plots as only have n =1 for it compared to n=3 for others.
