# Supplementary material for: FAK inhibition alone or in combination with adjuvant therapies reduces cancer stem cell activity
Source: NPJ Breast Cancer. 2021 May 28;7:65. doi: 10.1038/s41523-021-00263-3 (PMC8163772; doi:10.1038/s41523-021-00263-3)
Supplement: Supplementary file 2 — Reporting Summary [file 41523_2021_263_MOESM2_ESM.pdf]

nature research

Corresponding author(s): Nigel Bundred

Last updated by author(s): 11/09/2020

Reporting Summary

Nature Research wishes to improve the reproducibility of the work that we publish. This form provides structure for consistency and transparency in reporting. For further information on Nature Research policies, see our Editorial Policies and the Editorial Policy Checklist.

Statistics

For all statistical analyses, confirm that the following items are present in the figure legend, table legend, main text, or Methods section.

n/a	Confirmed	
☐	☒	The exact sample size (*n*) for each experimental group/condition, given as a discrete number and unit of measurement
☐	☒	A statement on whether measurements were taken from distinct samples or whether the same sample was measured repeatedly
☐	☒	The statistical test(s) used AND whether they are one- or two-sided<br>*Only common tests should be described solely by name; describe more complex techniques in the Methods section.*
☐	☒	A description of all covariates tested
☐	☒	A description of any assumptions or corrections, such as tests of normality and adjustment for multiple comparisons
☐	☒	A full description of the statistical parameters including central tendency (e.g. means) or other basic estimates (e.g. regression coefficient) AND variation (e.g. standard deviation) or associated estimates of uncertainty (e.g. confidence intervals)
☐	☒	For null hypothesis testing, the test statistic (e.g. *F*, *t*, *r*) with confidence intervals, effect sizes, degrees of freedom and *P* value noted<br>*Give P values as exact values whenever suitable.*
☒	☐	For Bayesian analysis, information on the choice of priors and Markov chain Monte Carlo settings
☒	☐	For hierarchical and complex designs, identification of the appropriate level for tests and full reporting of outcomes
☐	☒	Estimates of effect sizes (e.g. Cohen's *d*, Pearson's *r*), indicating how they were calculated

Our web collection on statistics for biologists contains articles on many of the points above.

Software and code

Policy information about availability of computer code

Data collection	Data was collected and analysed using SPSS 22.0 or Graph Pad prism 7.0
Data analysis	Data and graphs were analysed using Graph Pad Prism 7.0

For manuscripts utilizing custom algorithms or software that are central to the research but not yet described in published literature, software must be made available to editors and reviewers. We strongly encourage code deposition in a community repository (e.g. GitHub). See the Nature Research guidelines for submitting code & software for further information.

Data

Policy information about availability of data

All manuscripts must include a data availability statement. This statement should provide the following information, where applicable:

- Accession codes, unique identifiers, or web links for publicly available datasets
- A list of figures that have associated raw data
- A description of any restrictions on data availability

The datasets generated during and/or analysed during the current study are available from the corresponding author on reasonable request.

Field-specific reporting

Please select the one below that is the best fit for your research. If you are not sure, read the appropriate sections before making your selection.

☒ Life sciences ☐ Behavioural & social sciences ☐ Ecological, evolutionary & environmental sciences

For a reference copy of the document with all sections, see nature.com/documents/nr-reporting-summary-flat.pdf

Life sciences study design

All studies must disclose on these points even when the disclosure is negative.

Sample size	The sample size for the retrospective patient cohort was undertaken under the guidance of P Foden and J Morris statiscians at UHSM. 244 patrients were included.
Data exclusions	Only patients who had insufficient tissue on TMA were excluded from analysis.
Replication	We undertook multiple replicates of each experiment as detailed in the appropriate figures.
Randomization	n/a
Blinding	n/a

Reporting for specific materials, systems and methods

We require information from authors about some types of materials, experimental systems and methods used in many studies. Here, indicate whether each material, system or method listed is relevant to your study. If you are not sure if a list item applies to your research, read the appropriate section before selecting a response.

Materials & experimental systems

n/a	Involved in the study	
☐	☒	Antibodies
☒	☐	Eukaryotic cell lines
☒	☐	Palaeontology and archaeology
☐	☒	Animals and other organisms
☐	☒	Human research participants
☐	☒	Clinical data
☒	☐	Dual use research of concern

Methods

n/a	Involved in the study	
☒	☐	ChIP-seq
☐	☒	Flow cytometry
☒	☐	MRI-based neuroimaging

Antibodies

Antibodies used	7AAD (bioscience-6993), ALDH1 IF (Bioscience-611195), Aldefluor FACS (stem cell-01700), CK18 IF,IHC (Abcam-Ab668), pFAKTyr397 IHC (invitrogen 44-624), WB (CST 8556), tFAK IHC (Millipore 05-537) WB (CST 3285), GAPDH WB (CST 8444S), H2KD FACS (Biolegend 116615), ITGa6 IHC (Atlas HPA012696), Ki67 IHC (DAKO M7240),
Validation	For each antibody we ran a positive control and isotype negative control. for example ALDH Ab was validated on liver tissue, lymph gland for ITGa6, FAK - smooth muscle used as internal control and heart tissue.

Animals and other organisms

Policy information about studies involving animals; ARRIVE guidelines recommended for reporting animal research

Laboratory animals	NOD scid gamma (NSG) mice were utilised to evaluate the effects of FAK inhibition in vivo as they lack natural killer cells, B cells and T cells which would normally prevent the patient tissue from engrafting in the mouse. Prior to undertaking our in vivo work, we analysed expression pFAK using the method described in 2.2.3 and mRNA sequencing data to identify which of the 20 PDX models within the Breast Biology group over-expressed FAK.<br>Frozen chunks of tumour from triple negative PDX models RC37 and 193 were defrosted and implanted subcutaneously into 4 NSG mice of 6-8 weeks age. These tumours were then allowed to reach a combined size of 1250mm3. When the first mouse reached this size all the mice were culled, and the tumours harvested as 2x2mm chunks which were then intermixed and implanted subcutaneously into 20 NSG mice as outlined in supplementary figure 5A.
Wild animals	n/a

Field-collected samples	n/a
Ethics oversight	All procedures were performed in accordance with the Animals Scientific Procedures Act 1986 and approved by the UK home office. Project License 40/3645

Note that full information on the approval of the study protocol must also be provided in the manuscript.

Human research participants

Policy information about studies involving human research participants

Population characteristics	Twenty-seven primary Invasive Ductal Carcinoma samples were collected by the MCRC biobank. Patients were consented prior to surgery and samples taken from lesions with a pre-op size of >1.5cm (research ethics code 05/Q1403/159).
Recruitment	Human samples were recruited and consented by MCRC biobank.
Ethics oversight	research ethics code 05/Q1403/159

Note that full information on the approval of the study protocol must also be provided in the manuscript.

Clinical data

Policy information about clinical studies

All manuscripts should comply with the ICMJE guidelines for publication of clinical research and a completed CONSORT checklist must be included with all submissions.

Clinical trial registration	Not a clinical trial but ethical approval for the IDC cohort - REC 14/SW/1170
Study protocol	We can provide this on request.
Data collection	We collected 244 retrospective patient samples from surgery undertaken at uhsm between 2009-2010 across molecular phenotypes. The clinical data was retrieved from electronic records and patient notes and stored in anonymised way on a password protected SPSS database
Outcomes	To correlate FAK and CSC marker expression with clinical outcome.<br>Cohort make up was done with the guidance of statisticians P.Foden and J.Morris at UHSM.

Flow Cytometry

Plots

Confirm that:

☒ The axis labels state the marker and fluorochrome used (e.g. CD4-FITC).

☒ The axis scales are clearly visible. Include numbers along axes only for bottom left plot of group (a 'group' is an analysis of identical markers).

☒ All plots are contour plots with outliers or pseudocolor plots.

☒ A numerical value for number of cells or percentage (with statistics) is provided.

Methodology

Sample preparation	Single cells were isolated and then analysed for ALDH using the Aldefluor assay from Stemcell technologies. The protocol was carried out as per the manufacturer's guidelines with 1 million cells per analysis condition. For every test sample a separate sample was created which contains the cell suspension and 5l of DEAB inhibitor. Cells were suspended as 1 million cells per 1ml of Aldefluor buffer, to which 5ul of BAA (Bodipy-aminoacetaldehyde was added) was added. 0.5mls of this solution was then added to a 1.5ml Eppendorf containing 5ul of DEAB. Samples were incubated for 45 minutes at 37oC. Following incubation, samples were centrifuged at 250g for 5 minutes and the supernatant removed prior to resuspension in 0.5mls of Aldefluor buffer. When re-suspended 5l per tube of 7AAD was added as a live dead stain. If PDX tumours were being analysed 1ul per tube of H2KD was added to exclude mouse cells. These antibodies were then incubated for a further 10 minutes on ice prior to analysis.<br>Cells were then either sorted or analysed. When expression was being analysed the Novocyte and LSR II flow cytometers were used. The Aldefluor assay used the 488nm laser for excitation and the 515-545nm channel for detection. The 7AAD was excited using the 488nm laser used and detected using the 650-670nm channel. The H2KD conjugated with pacific blue was excited using the 405nm laser and detected using the 425-475nm channel for detection.<br>A minimum cell count of 200,000 was used. Data was exported as an FCS file for analysis on Flow Jo.<br>When cells were flow sorted this was done on the Influx and supervised by Antonio Banyard of the Flow cytometry core facilities team at the MCRC. Figure 2.2F demonstrates how ALDH+ and ALDH low expressing cells were sorted. ALDH+ expression was defined relative to a 0.1% DEAB gate. Aldefluor low expressing cells were also isolated as the bottom 20% of Aldefluor expression and gated as demonstrated in red in figure 2.2F. These samples were collected in FACs buffer and spun down and lysed for western blot analysis.

Instrument	When expression was being analysed the Novocyte and LSR II flow cytometers were used.
Software	Data was exported as an FCS file for analysis on Flow Jo.
Cell population abundance	A minimum cell count of 200,000 was used.
Gating strategy	When cells were just analysed they were gated as demonstrated in figure 6H.<br>When cells were flow sorted this was done on the Influx and supervised by Antonio Banyard of the Flow cytometry core facilities team at the MCRC. ALDH+ expression was defined relative to a 0.1% DEAB gate. Aldefluor low expressing cells were also isolated as the bottom 20% of Aldefluor expression and gated as demonstrated in supplementary figure 1C. These samples were collected in FACs buffer and spun down and lysed for western blot analysis.

☒ Tick this box to confirm that a figure exemplifying the gating strategy is provided in the Supplementary Information.
